# Parameter Estimation with Data-Driven Nonparametric Likelihood Functions

**DOI:** 10.3390/e21060559

**Published:** 2019-06-03

**Authors:** Shixiao W. Jiang, John Harlim

**Affiliations:** 1 Department of Mathematics, the Pennsylvania State University, 109 McAllister Building, University Park, PA 16802-6400, USA; 2 Department of Meteorology and Atmospheric Science, the Pennsylvania State University, 503 Walker Building, University Park, PA 16802-5013, USA; 3 Institute for CyberScience, the Pennsylvania State University, 224B Computer Building, University Park, PA 16802, USA

**Keywords:** Bayesian inference, MCMC, diffusion maps, nonparametric likelihood function, surrogate modeling, reproducing kernel Hilbert space, kernel embedding of the conditional distribution

## Abstract

In this paper, we consider a surrogate modeling approach using a data-driven nonparametric likelihood function constructed on a manifold on which the data lie (or to which they are close). The proposed method represents the likelihood function using a spectral expansion formulation known as the kernel embedding of the conditional distribution. To respect the geometry of the data, we employ this spectral expansion using a set of data-driven basis functions obtained from the diffusion maps algorithm. The theoretical error estimate suggests that the error bound of the approximate data-driven likelihood function is independent of the variance of the basis functions, which allows us to determine the amount of training data for accurate likelihood function estimations. Supporting numerical results to demonstrate the robustness of the data-driven likelihood functions for parameter estimation are given on instructive examples involving stochastic and deterministic differential equations. When the dimension of the data manifold is strictly less than the dimension of the ambient space, we found that the proposed approach (which does not require the knowledge of the data manifold) is superior compared to likelihood functions constructed using standard parametric basis functions defined on the ambient coordinates. In an example where the data manifold is not smooth and unknown, the proposed method is more robust compared to an existing polynomial chaos surrogate model which assumes a parametric likelihood, the non-intrusive spectral projection. In fact, the estimation accuracy is comparable to direct MCMC estimates with only eight likelihood function evaluations that can be done offline as opposed to 4000 sequential function evaluations, whenever direct MCMC can be performed. A robust accurate estimation is also found using a likelihood function trained on statistical averages of the chaotic 40-dimensional Lorenz-96 model on a wide parameter domain.

## 1. Introduction

Bayesian inference is a popular approach for solving inverse problems with far-reaching applications, such as parameter estimation and uncertainty quantification (see for example [1,2,3]). In this article, we will focus on a classical Bayesian inference problem of estimating the conditional distribution of hidden parameters of dynamical systems from a given set of noisy observations. In particular, let x(t;θ) be a time-dependent state variable, which implicitly depends on the parameter θ through the following initial value problem,

(1) $$\dot{x}=f\left(x,\theta \right),\;\;\;x\left(0\right)=x_{0}.$$

Here, for any fixed θ, *f* can be either deterministic or stochastic. Our goal is to estimate the conditional distribution of θ, given discrete-time noisy observations $y^{†}=\left\{y_{1}^{†},\ldots ,y_{T}^{†}\right\}$, where:

(2) $$y_{i}^{†}=g\left(x_{i}^{†},\xi_{i}\right),\;\;\;i=1,\ldots ,T.$$

Here, $x_{i}^{†}\equiv x\left(t_{i};\theta^{†}\right)$ are the solutions of Equation (1) for a specific hidden parameter $\theta^{†}$, *g* is the observation function, and $\xi_{i}$ are unbiased noises representing the measurement or model error. Although the proposed approach can also estimate the conditional density of the initial condition $x_{0}$, we will not explore this inference problem in this article.

Given a prior density, $p_{0}\left(\theta \right)$, Bayes’ theorem states that the conditional distribution of the parameter θ can be estimated as,

(3) $$p\left(\theta |y^{†}\right)\propto p\left(y^{†}|\theta \right)p_{0}\left(\theta \right),$$

where $p(y^{†}|\theta )$ denotes the likelihood function of θ given the measurements $y^{†}$ that depend on a hidden parameter value $\theta^{†}$ through (2). In most applications, the statistics of the conditional distribution $p(\theta |y^{†})$ are the quantity of interest. For example, one can use the mean statistic as a point estimator of $\theta^{†}$ and the higher order moments for uncertainty quantification. To realize this goal, one draws samples of $p(\theta |y^{†})$ and estimates these statistics via Monte Carlo averages over these samples. In this application, Markov Chain Monte Carlo (MCMC) is a natural sampling method that plays a central role in the computational statistics behind most Bayesian inference techniques [4].

In our setup, we assume that for any θ, one can simulate:

(4) $$\begin{array}{l} y_{i}\left(\theta \right)=g\left(x_{i}\left(\theta \right),\xi_{i}\right),i=1,\ldots ,T. \end{array}$$

where $x_{i}\left(\theta \right)\equiv x\left(t_{i};\theta \right)$ denote solutions to the initial value problem in Equation (1). If the observation function has the following form,

(5) $$\begin{array}{l} g\left(x_{i}\left(\theta \right),\xi_{i}\right)=h\left(x_{i}\left(\theta \right)\right)+\xi_{i}, \end{array}$$

where $\xi_{i}$ are i.i.d. noises, then one can define the likelihood function of θ, $p(y^{†}|\theta )$, as a product of the density functions of the noises $\xi_{i}$,

(6) $$p\left(y^{†}|\theta \right)\equiv \prod_{i=1}^{T}p\left(\xi_{i}\right)=\prod_{i=1}^{T}p\left(y_{i}^{†}-h\left(x_{i}\left(\theta \right)\right)\right).$$

When the observations are noise-less, $\xi_{i}=0$, and the underlying system is an Itô diffusion process with additive or multiplicative noises, one can use the Bayesian imputation to approximate the likelihood function [5]. In both parametric approaches, it is worth noting that the dependence of the likelihood function on the parameter is implicit through the solutions $x_{i}\left(\theta \right)$. Practically, this implicit dependence is the source of the computational burden in evaluating the likelihood function since it requires solving the dynamical model in (1) or every proposal in the MCMC chain. In the case when simulating $y_{i}\left(\theta \right)$ is computationally feasible, but the likelihood function is intractable, then one can use, e.g., the Approximate Bayesian Computation (ABC) rejection algorithm [6,7] for Bayesian inference. Basically, the ABC rejection scheme generates the samples of $p(\theta |y^{†})$ by comparing the simulated $y_{i}\left(\theta \right)$ to the observed data, $y_{i}^{†}$, with an appropriate choice of metric comparison for each proposal $\theta ∼p_{0}\left(\theta \right)$. In general, however, repetitive evaluation of (4) can be expensive when the dynamics in (1) is high-dimensional and/or stiff, or when *T* is large, or when the function *g* is an average of a long time series. Our goal is to address this situation in addition to not knowing the approximate likelihood function.

Broadly speaking, the existing approaches to overcome repetitive evaluation of (4) require knowledge of an approximate likelihood function such as in (6). They can be grouped into two classes. The first class consists of methods that improve/accelerate the sampling strategy; for example, the Hamiltonian Monte Carlo [8], adaptive MCMC [9], and delay rejection adaptive Metropolis [10], just to name a few. The second class consists of methods that avoid solving the dynamical model in (1) when running the MCMC chain by replacing it with a computationally more efficient model on a known parameter domain. This class of approach, also known as *surrogate modeling*, includes Gaussian process models [11], polynomial chaos [12,13], and enhanced model error [14]; for example, the non-intrusive spectral projection [13] approximate $x_{i}\left(\theta \right)$ in (6) with a polynomial chaos expansion. Another related approach, which also avoids MCMC on top of integrating (1), is to employ a polynomial expansion on the likelihood function [15]. This method represents the parametric likelihood function in (6) with orthonormal basis functions of a Hilbert space weighted by the prior measure. This choice of basis functions makes the computation for the statistics of the posterior density straightforward, and thus, MCMC is not needed.

In this paper, we consider a surrogate modeling approach where a nonparametric likelihood function is constructed using a data-driven spectral expansion. By nonparametric, we mean that our approach does not require any parametric form or assume any distribution as in (6). Instead, we approximate the likelihood function using the kernel embedding of conditional distribution formulation introduced in [16,17]. In our application, we will extend their formulation onto a Hilbert space weighted by the sampling measure of the training dataset as in [18]. We will rigorously demonstrate that using orthonormal basis functions of this data-driven weighted Hilbert space, the error bound is independent of the variance of the basis functions, which allows us to determine the amount of training data for accurate likelihood function estimations.

Computationally, assuming that the observations lie on (or close to) a Riemannian manifold N embedded in $R^{n}$ with sampling density q(y), we apply the diffusion maps algorithm [19,20] to approximate orthonormal basis functions $\varphi_{k}\in L^{2}\left(N,q\right)$ using the training dataset. Subsequently, a nonparametric likelihood function is represented as a weighted sum of these data-driven basis functions, where the coefficients are precomputed using the kernel embedding formulation. In this fashion, our approach respects the geometry of the data manifold. Using this nonparametric likelihood function, we then generate the MCMC chain for estimating the conditional distribution of hidden parameters. For the present work, our aim is to demonstrate that one can obtain accurate and robust parameter estimation by implementing a simple Bayesian inference algorithm, the Metropolis scheme, with the data-driven nonparametric likelihood function. We should also point out that the present method is computationally feasible on low-dimensional parameter space, like any other surrogate modeling approach. Possible ways to overcome this dimensionality issue will be discussed.

This paper is organized as follows: In Section 2, we review the formulation of the reproducing kernel Hilbert space to estimate conditional density functions. In Section 3, we discuss the error estimate of the likelihood function approximation. In Section 4, we discuss the construction of the analytic basis functions for the Euclidean data manifold, as well as the data-driven basis functions with the diffusion maps algorithm for data that lie on embedded Riemannian geometry. In Section 5, we provide numerical results with parameter estimation application on instructive examples. In one of the examples where the dynamical model is low-dimensional and the observation is in the form of (5), we compare the proposed approach with the direct MCMC and non-intrusive spectral projection method (both schemes use likelihood of the form (6)). In addition, we will also demonstrate the robustness of the proposed approach on an example where *g* is a statistical average of a long-time trajectory (in which the likelihood is intractable) and the dynamical model has relatively high-dimensional chaotic dynamics such that repetitive evaluation of (4) is numerically expensive. In Section 6, we conclude this paper with a short summary. We accompany this paper with Appendices for treating large amount of data and more numerical results.

## 2. Conditional Density Estimation via Reproducing Kernel Weighted Hilbert Spaces

Let $y\in N\subseteq R^{n}$, where N is a smooth manifold with intrinsic dimension d≤n. In practice, we measure the observations in the ambient coordinates and denote their components as $y=\{y^{1},\ldots ,y^{n}\}$. For the parameter θ space, M has a Euclidean structure with components, $\theta =\{\theta^{1},\ldots ,\theta^{m}\}$, so M is assumed to be either an *m*-dimensional hyperrectangle or $R^{m}$. For training, we are given *M* number of training parameters ${\left\{\theta_{j}\right\}}_{j=1,\ldots ,M}={\left\{\theta_{j}^{1},\ldots ,\theta_{j}^{m}\right\}}_{j=1,\ldots ,M}$. For each training parameter $\theta_{j}$, we generate a discrete time series of length *N* for noisy observation data $\left\{y_{i,j}\right\}=\left\{y_{i,j}^{1},\ldots ,y_{i,j}^{n}\right\}\in R^{n}$ for i=1,…,N, and j=1,…,M. Here, the sub-index *i* and the sub-index *j* of $y_{i,j}$ correspond to the $i^{\mathrm{th}}$ observation data for the $j^{\mathrm{th}}$ training parameter $\theta_{j}$. Our goal for training is to learn the conditional density p(y|θ) from the training dataset ${\left\{\theta_{j}\right\}}_{j=1,\ldots ,M}$ and ${\left\{y_{i,j}\right\}}_{j=1,\ldots ,M}^{i=1,\ldots ,N}$ for arbitrary y and θ within the range of ${\left\{\theta_{j}\right\}}_{j=1,\ldots ,M}$.

The construction of the conditional density p(y|θ) is based on a machine learning tool known as the kernel embedding of the conditional distribution formulation introduced in [16,17]. In their formulation, the representation of conditional distributions is an element of a Reproducing Kernel Hilbert Space (RKHS).

Recently, the representation using a Reproducing Kernel Weighted Hilbert Space (RKWHS) was introduced in [18]. That is, let $\Psi_{k}:=\psi_{k}q$ be the orthonormal basis of $L^{2}\left(N,q^{-1}\right)$, where they are eigenbasis of an integral operator,

(7) $$\begin{array}{l} Kf\left(y\right)=\int_{N}K\left(y,y^{\prime }\right)f\left(y^{\prime }\right)q^{-1}\left(y^{\prime }\right)dV,\;f\in L^{2}\left(N,q^{-1}\right), \end{array}$$

that is, $K\Psi_{k}=\lambda_{k}\Psi_{k}$.

In the case where N is compact and K is Hilbert–Schmidt, the kernel can be written as,

(8) $$\begin{array}{l} K\left(y,y^{\prime }\right)=\sum_{k=1}^{\infty }\lambda_{k}\Psi_{k}\left(y\right)\Psi_{k}\left(y^{\prime }\right), \end{array}$$

which converges in $L^{2}\left(N,q^{-1}\right)$. Define the feature map $\Phi :M\to \ell_{2}$ as,

(9) $$\begin{array}{l} \Phi \left(y\right):=\left\{\Phi_{k}\left(y\right)={\sqrt{\lambda }}_{k}\Psi_{k}\left(y\right):k\in Z^{+},y\in N\right\}. \end{array}$$

Therefore, any $f\in L^{2}\left(N,q^{-1}\right)$ can be represented as $f=\sum_{k=1}^{\infty }{\hat{f}}_{k}\Psi_{k}=\sum_{k=1}^{\infty }\frac{\hat{f_{k}}}{\sqrt{\lambda_{k}}}\Phi_{k}$, where ${\hat{f}}_{k}={\langle f,\Psi_{k}\rangle }_{q^{-1}}=\langle f,\psi_{k}\rangle :=\int_{N}f\left(y\right)\psi_{k}\left(y\right)dV$ and provided that $\sum_{k}{\left|{\hat{f}}_{k}\right|}^{2}/\lambda_{k}<\infty$. If we define ${\langle f,g\rangle }_{H_{q^{-1}}}:=\sum_{k=1}^{\infty }\frac{\hat{f_{k}}\hat{g_{k}}}{\lambda_{k}}$, we can write the kernel in (8) as $K\left(y,y^{\prime }\right)={\langle \Phi \left(y\right),\Phi \left(y^{\prime }\right)\rangle }_{H_{q^{-1}}}$. Throughout this manuscript, we denote the RKHS $H_{q^{-1}}\left(N\right)$ generating the feature map Φ in (9) as the space of square integrable functions with a reproducing property,

$$\begin{array}{lll} f(y) & = & {\langle f,K\left(\cdot ,y\right)\rangle }_{H_{q^{-1}}}:=\sum_{k=1}^{\infty }\frac{{\hat{f}}_{k}{\langle K\left(\cdot ,y\right),\Psi_{k}\rangle }_{q^{-1}}}{\lambda_{k}}=\sum_{k=1}^{\infty }\frac{{\hat{f}}_{k}}{\sqrt{\lambda_{k}}}\Phi_{k}\left(y\right)={\langle f,\Phi \left(y\right)\rangle }_{H_{q^{-1}}},\;\forall y\in N, \end{array}$$

induced by the basis of $\Psi_{k}\in L^{2}\left(N,q^{-1}\right)$. While this definition deceptively suggests that $H_{q^{-1}}\left(N\right)$ is similar to $L^{2}\left(N,q^{-1}\right)$, we should also point out that the RKHS requires that the Dirac functional $\delta_{x}:H_{q^{-1}}\left(N\right)\to R$ defined as $\delta_{x}f=f\left(x\right)$ be continuous. Since $L^{2}$ contains a class of functions, it is not an RKHS and $H_{q^{-1}}\left(N\right)\subset L^{2}\left(N,q^{-1}\right)$. See, e.g., Chapter 4 of [21] for more details. Using the same definition, we denote $H_{{\tilde{q}}^{-1}}\left(M\right)$ as the RKHS induced by orthonormal basis of $L^{2}\left(M,{\tilde{q}}^{-1}\right)$ of functions of the parameter θ.

In this work, we will represent conditional density functions using the RKWHS induced by the data, where the bases will be constructed using the diffusion maps algorithm. The outcome of the training is an estimate of the conditional density, $\hat{p}\left(y|\theta \right)$, for arbitrary y and θ within the range of ${\left\{\theta_{j}\right\}}_{j=1,\ldots ,M}$.

### 2.1. Review of Nonparametric RKWHS Representation of Conditional Density Functions

We first review the RKWHS representation of conditional density functions deduced in [18]. Let $\psi_{k}\left(y\right)$ be the orthonormal basis functions of $L^{2}\left(N,q\right)$, where N contains the domain of the training data $y_{i,j}$, and the weight function $q\left(y\right)$ is defined with respect to the volume form inherited by N from the ambient space $R^{n}$. Let $\varphi_{l}\left(\theta \right)\in L^{2}\left(M,\tilde{q}\right)$ be the orthonormal basis functions in the parameter θ space, where the training parameters are $\theta_{j}\in M$, with weight function $\tilde{q}\left(\theta \right)$. For finite modes, $k=1,\ldots ,K_{1}$, and $l=1,\ldots ,K_{2}$, a nonparametric RKWHS representation of the conditional density can be written as follows [18]:

(10) $$\hat{p}\left(y|\theta \right)=\sum_{k=1}^{K_{1}}{\hat{c}}_{Y|\theta ,k}\psi_{k}\left(y\right)q\left(y\right),$$

where $\hat{p}\left(y|\theta \right)$ denotes an estimate of the conditional density $p\left(y|\theta \right)\in H_{q^{-1}}\left(N\right)$, and the expansion coefficients are defined as:

(11) $${\hat{c}}_{Y|\theta ,k}=\sum_{l=1}^{K_{2}}{\left[C_{Y\Theta }C_{\Theta \Theta }^{-1}\right]}_{kl}\varphi_{l}\left(\theta \right).$$

Here, the matrix $C_{Y\Theta }$ is $K_{1}\times K_{2}$, and the matrix $C_{\Theta \Theta }$ is $K_{2}\times K_{2}$, whose components can be approximated by Monte Carlo averages [18]:

(12) $$\begin{array}{lll} {\left[C_{Y\Theta }\right]}_{ks} & = & E_{Y\Theta }\left[\psi_{k}\varphi_{s}\right]\approx \frac{1}{MN}\sum_{j=1}^{M}\sum_{i=1}^{N}\psi_{k}\left(y_{i,j}\right)\varphi_{s}\left(\theta_{j}\right), \end{array}$$

(13) $$\begin{array}{lll} {\left[C_{\Theta \Theta }\right]}_{sl} & = & E_{\Theta \Theta }\left[\varphi_{s}\varphi_{l}\right]\approx \frac{1}{M}\sum_{j=1}^{M}\varphi_{s}\left(\theta_{j}\right)\varphi_{l}\left(\theta_{j}\right), \end{array}$$

where the expectations E are taken with respect to the sampling densities of the training dataset ${\left\{y_{i,j}\right\}}_{j=1,\ldots ,M}^{i=1,\ldots ,N}$ and ${\left\{\theta_{j}\right\}}_{j=1,\ldots ,M}$. The equation for the expansion coefficients in Equation (11) is based on the theory of kernel embedding of the conditional distribution [16,17,18]. See [18] for the detailed proof of Equations (11)–(13). Note that for RKWHS representation, the weight functions *q* and $\tilde{q}$ can be different from the sampling densities of the training dataset ${\left\{y_{i,j}\right\}}_{j=1,\ldots ,M}^{i=1,\ldots ,N}$ and ${\left\{\theta_{j}\right\}}_{j=1,\ldots ,M}$, respectively. This generalizes the representation in [18], which sets the weights *q* and $\tilde{q}$ to be the sampling densities of the training dataset $\left\{y_{i,j}\right\}$ and $\left\{\theta_{j}\right\}$, respectively. If the assumption of $p\left(y|\theta \right)\in H_{q^{-1}}\left(N\right)$ is not satisfied, then $C_{\Theta \Theta }$ can be singular. In such a case, one can follow the suggestion in [16,17] to regularize the linear regression in (11) by replacing $C_{\Theta \Theta }^{-1}$ with ${\left(C_{\Theta \Theta }+\lambda I_{K_{2}}\right)}^{-1}$, where λ∈R is an empirically-chosen parameter and $I_{K_{2}}$ denotes an identity matrix of size $K_{2}\times K_{2}$.

Incidentally, it is worth mentioning that the conditional density in (10) and (11) is represented as a regression in infinite-dimensional spaces with basis functions $\psi_{k}\left(y\right)$ and $\varphi_{l}\left(\theta \right)$. The expression (10) is a nonparametric representation in the sense that we do not assume any particular distribution for the density function $p\left(y|\theta \right)$. In this representation, only training dataset ${\left\{y_{i,j}\right\}}_{j=1,\ldots ,M\;}^{i=1,\ldots ,N}$ and ${\left\{\theta_{j}\right\}}_{j=1,\ldots ,M}$ with appropriate basis functions are used to specify the coefficients ${\hat{c}}_{Y|\theta ,k}$ and the densities $\hat{p}\left(y|\theta \right)$. In Section 4, we will demonstrate how to construct the appropriate basis completely from the training data, motivated by the theoretical result in Section 3 below.

### 2.2. Simplification of the Expansion Coefficients (11)

If the weight function $\tilde{q}\left(\theta \right)$ is the sampling density of the training parameters ${\left\{\theta_{j}\right\}}_{j=1,\ldots ,M}$, the matrix $C_{\Theta \Theta }$ in (13) can be simplified to a $K_{2}\times K_{2}$ identity matrix,

(14) $${\left[C_{\Theta \Theta }\right]}_{sl}=E_{\Theta \Theta }\left[\varphi_{s}\varphi_{l}\right]=\int_{M}\varphi_{s}\left(\theta \right)\varphi_{l}\left(\theta \right)\tilde{q}\left(\theta \right)d\theta =\delta_{sl}.$$

where $\delta_{sl}$ is the Kronecker delta function. Here, the second equality follows from the weight $\tilde{q}\left(\theta \right)$ being the sampling density, and the third equality follows from the orthonormality of $\varphi_{l}\left(\theta \right)\in L^{2}\left(M,\tilde{q}\right)$ with respect to the weight function $\tilde{q}$. Then, the expansion coefficients ${\hat{c}}_{Y|\theta ,k}$ in (11) can be simplified to,

(15) $${\hat{c}}_{Y|\theta ,k}=\sum_{l=1}^{K_{2}}{\left[C_{Y\Theta }\right]}_{kl}\varphi_{l}\left(\theta \right),$$

with the $K_{1}\times K_{2}$ matrix $C_{Y\Theta }$ still given by (12). In this work, we always take the weight function $\tilde{q}\left(\theta \right)$ to be the sampling density of the training parameters ${\left\{\theta_{j}\right\}}_{j=1,\ldots ,M}$ for the simplification of the expansion coefficients ${\hat{c}}_{Y|\theta ,k}$ in (15). This assumption is not too restrictive since the training parameters are specified by the users.

Finally, the formula in (10) combined with the expansion coefficients ${\hat{c}}_{Y|\theta ,k}$ in (15) and the matrix $C_{Y\Theta }$ in (12) forms an RKWHS representation of the conditional density $p\left(y|\theta \right)$ for arbitrary y and θ. Numerically, the training outcome is the matrix $C_{Y\Theta }$ in (12), and then, the conditional density $\hat{p}\left(y|\theta \right)$ can be represented by (10) with coefficients (15) using the basis functions ${\left\{\psi_{k}\left(y\right)\right\}}_{k=1}^{K_{1}}$ and ${\left\{\varphi_{l}\left(\theta \right)\right\}}_{l=1}^{K_{2}}$. From above, one can see that two important questions naturally arise as a consequence of the usage of RKWHS representation: first, whether the representation $\hat{p}\left(y|\theta \right)$ in (10) is valid in estimating the conditional density $p\left(y|\theta \right)$; second, how to construct the orthonormal basis functions $\psi_{k}\left(y\right)\in L^{2}\left(N,q\right)$ and $\varphi_{l}\left(\theta \right)\in L^{2}\left(M,\tilde{q}\right)$. We will address these two important questions in the next two sections.

## 3. Error Estimation

In this section, we focus on the error estimation of the expansion coefficient ${\hat{c}}_{Y|\theta_{j},k}$ and, later, the conditional density $\hat{p}\left(y|\theta_{j}\right)$ at the training parameter $\theta_{j}$. The notation ${\hat{c}}_{Y|\theta_{j},k}$ is defined as the expansion coefficient ${\hat{c}}_{Y|\theta ,k}$ in (15), evaluated at the training parameter $\theta_{j}$. Let the total number of basis functions in parameter space, $K_{2}$, be equal to the total number of training parameters, *M*, that is, $K_{2}=M$. Denoting $\Phi =\left[{\vec{\varphi }}_{1},\ldots ,{\vec{\varphi }}_{M}\right]\in R^{M\times M}$, where the $j^{\mathrm{th}}$ component of ${\vec{\varphi }}_{l}$ approximates the basis function evaluated at the training data $\varphi_{l}\left(\theta_{j}\right)$, we can write the last equality in (14) in a compact form as $M^{-1}\Phi^{⊤}\Phi =I_{M}$. This also means that, $M^{-1}\Phi \Phi^{⊤}=I_{M}$, the components of which are,

(16) $$\frac{1}{M}\sum_{l=1}^{M}\varphi_{l}\left(\theta_{s}\right)\varphi_{l}\left(\theta_{j}\right)=\delta_{sj}.$$

For the training parameter $\theta_{j}$, we can simplify the expansion coefficient ${\hat{c}}_{Y|\theta_{j},k}$ by substituting Equation (12) into Equation (15),

(17) $$\begin{array}{l} {\hat{c}}_{Y|\theta_{j},k}=\sum_{l=1}^{M}{\left[C_{Y\Theta }\right]}_{kl}\varphi_{l}\left(\theta_{j}\right)\approx \sum_{l=1}^{M}\left[\frac{1}{MN}\sum_{s=1}^{M}\sum_{i=1}^{N}\psi_{k}\left(y_{i,s}\right)\varphi_{l}\left(\theta_{s}\right)\right]\varphi_{l}\left(\theta_{j}\right)=\frac{1}{N}\sum_{i=1}^{N}\psi_{k}\left(y_{i,j}\right), \end{array}$$

where the last equality follows from (16).

### 3.1. Error Estimation Using Arbitrary Bases

We first study the error estimation for the expansion coefficient ${\hat{c}}_{Y|\theta_{j},k}$. For each training parameter $\theta_{j}$, the conditional density function $p\left(y|\theta_{j}\right)\in H_{q^{-1}}\left(N\right)$ can be analytically represented in the form,

(18) $$p\left(y|\theta_{j}\right)=\sum_{k=1}^{\infty }c_{Y|\theta_{j},k}\psi_{k}\left(y\right)q\left(y\right),$$

due to the completeness of $L^{2}\left(N,q\right)$. Here, the analytic expansion coefficient $c_{Y|\theta_{j},k}$ is given by,

(19) $$c_{Y|\theta_{j},k}=\langle p\left(\cdot |\theta_{j}\right),\psi_{k}\rangle .$$

Note that the estimator ${\hat{c}}_{Y|\theta_{j},k}$ in (17) is a Monte Carlo approximation of the expansion coefficient $c_{Y|\theta_{j},k}$ in (19), i.e.,

(20) $$c_{Y|\theta_{j},k}=\langle p\left(\cdot |\theta_{j}\right),\psi_{k}\rangle =E_{Y|\theta_{j}}\left[\psi_{k}\left(Y\right)\right]\approx \frac{1}{N}\sum_{i=1}^{N}\psi_{k}\left(y_{i,j}\right),$$

where the last equality follows from the training dataset ${\left\{y_{i,j}\right\}}_{i=1,\ldots ,N}$, which admits a conditional density $p\left(y|\theta_{j}\right)$. Note also that in the following theorems and propositions, the condition $p\left(y|\theta_{j}\right)\in H_{q^{-1}}\left(N\right)$ is required. In Section 5.2 and Appendix B, we will provide an example to discuss this condition in detail. Next, we provide the unbiasedness and consistency of the estimator ${\hat{c}}_{Y|\theta_{j},k}$.

**Proposition** **1.** *Let ${\left\{y_{i,j}\right\}}_{i=1,\ldots ,N}$ be i.i.d. samples of $Y|\theta_{j}$ with density $p(y|\theta_{j})$. Let $p\left(y|\theta_{j}\right)\in H_{q^{-1}}\left(N\right)$ and $\left\{\psi_{k}\left(y\right)\right\}$ form a complete orthonormal basis of $L^{2}\left(N,q\right)$. Assume that ${\mathrm{Var}}_{Y|\theta_{j}}\left[\psi_{k}\left(Y\right)\right]$ is finite, then ${\hat{c}}_{Y|\theta_{j},k}$ defined in (17) is an unbiased and consistent estimator for $c_{Y|\theta_{j},k}$ in (19).*

**Proof.**  The estimator ${\hat{c}}_{Y|\theta_{j},k}$ is unbiased,

(21) $$E{\hat{c}}_{Y|\theta_{j},k}=\frac{1}{N}\sum_{i=1}^{N}E_{Y|\theta_{j}}\psi_{k}\left(Y_{i,j}\right)=c_{Y|\theta_{j},k}.$$

where the expectation is taken with respect to the conditional density $p\left(y|\theta_{j}\right)$. If the variance, Var${}_{Y|\theta_{j}}\left[\psi_{k}\left(Y\right)\right]$, is finite, then the variance of ${\hat{c}}_{Y|\theta_{j},k}$ converges to zero as the number of training data N→∞,

(22) $$\mathrm{Var}\left({\hat{c}}_{Y|\theta_{j},k}\right)=\frac{1}{N}{\mathrm{Var}}_{Y|\theta_{j}}\left[\psi_{k}\left(Y\right)\right]\to 0,\;\;\mathrm{as}\;N\to \infty .$$

Then, we can obtain that the estimator ${\hat{c}}_{Y|\theta_{j},k}$ is consistent,

$$P_{r}\left(\left|{\hat{c}}_{Y|\theta_{j},k}-c_{Y|\theta_{j},k}\right|>\varepsilon \right)\leq \frac{\mathrm{Var}\left({\hat{c}}_{Y|\theta_{j},k}\right)}{\varepsilon^{2}}\to 0,\;\mathrm{as}\;N\to \infty ,\;\mathrm{for}\;\forall \varepsilon >0,$$

where Chebyshev’s inequality has been used. □

If the estimator of $p(y|\theta_{j})$ is given by the representation with an infinite number of basis functions, $\tilde{p}\left(y|\theta_{j}\right)=\sum_{k=1}^{\infty }{\hat{c}}_{Y|\theta_{j},k}\psi_{k}\left(y\right)q\left(y\right)$, then the estimator $\tilde{p}\left(y|\theta_{j}\right)$ is pointwise unbiased for every observation y. However, in the numerical implementation, only a finite number of basis functions can be used in the representation (10). Numerically, the estimator of $p(y|\theta_{j})$ is given by the representation (10) at the training parameter $\theta_{j}$,

$$\hat{p}\left(y|\theta_{j}\right)=\sum_{k=1}^{K_{1}}{\hat{c}}_{Y|\theta_{j},k}\psi_{k}\left(y\right)q\left(y\right).$$

Then, the pointwise error of the estimator, $\hat{e}\left(y|\theta_{j}\right)$, can be defined as:

(23) $$\begin{array}{lll} \hat{e}\left(y|\theta_{j}\right) & \equiv & p\left(y|\theta_{j}\right)-\hat{p}\left(y|\theta_{j}\right)\\ & = & \sum_{k=K_{1}+1}^{\infty }c_{Y|\theta_{j},k}\psi_{k}\left(y\right)q\left(y\right)+\sum_{k=1}^{K_{1}}\left(c_{Y|\theta_{j},k}-{\hat{c}}_{Y|\theta_{j},k}\right)\psi_{k}\left(y\right)q\left(y\right).\; \end{array}$$

It can be seen that the estimator $\hat{p}\left(y|\theta_{j}\right)$ is no longer unbiased or consistent due to the first error term in (23) induced by modes $k>K_{1}$. Next, we estimate the expectation and the variance of an $L^{2}$-norm error of $\hat{p}\left(y|\theta_{j}\right)$ for all training parameters $\theta_{j}$.

**Theorem** **1.** *Let the condition in Proposition 1 be satisfied for all ${\left\{\theta_{j}\right\}}_{j=1,\ldots ,M}$, and Var${}_{Y|\theta_{j}}\left[\psi_{k}\left(Y\right)\right]$ be finite for all $k\in N^{+}$. Define the $L^{2}$-norm error,*

(24) $${\hat{e}}_{L^{2}}={\left(\sum_{j=1}^{M}\int_{N}{\left|\hat{e}\left(y|\theta_{j}\right)\right|}^{2}q^{-1}\left(y\right)dV\right)}^{1/2},$$

*where $\hat{e}\left(y|\theta_{j}\right)$ is the pointwise error in (23), and dV is the volume form inherited by the manifold N from the ambient space $R^{n}$ [18,20]. Then,*

(25) $$\begin{array}{lll} E\left[{\hat{e}}_{L^{2}}\right] & \leq & {\left(\sum_{j=1}^{M}\sum_{k=K_{1}+1}^{\infty }{\left[c_{Y|\theta_{j},k}\right]}^{2}+\frac{1}{N}\sum_{j=1}^{M}\sum_{k=1}^{K_{1}}{\mathrm{Var}}_{Y|\theta_{j}}\left[\psi_{k}\left(Y\right)\right]\right)}^{\frac{1}{2}}, \end{array}$$

(26) $$\begin{array}{lll} \mathrm{Var}\left[{\hat{e}}_{L^{2}}\right] & \leq & \sum_{j=1}^{M}\sum_{k=K_{1}+1}^{\infty }{\left[c_{Y|\theta_{j},k}\right]}^{2}+\frac{1}{N}\sum_{j=1}^{M}\sum_{k=1}^{K_{1}}{\mathrm{Var}}_{Y|\theta_{j}}\left[\psi_{k}\left(Y\right)\right], \end{array}$$

*where E and Var are defined with respect to the joint distribution of $p(y|\theta_{j})$ for all ${\left\{\theta_{j}\right\}}_{j=1,\ldots ,M}$. Moreover, $E\left[{\hat{e}}_{L^{2}}\right]$ and Var$\left[{\hat{e}}_{L^{2}}\right]$ converge to zero as $K_{1}\to \infty$ and then N→∞, where the limiting operations of $K_{1}$ and N are not commutative.*

**Proof.**  The expectation of ${\hat{e}}_{L^{2}}$ can be estimated as,

(27) $$\begin{array}{lll} {\left(E\left[{\hat{e}}_{L^{2}}\right]\right)}^{2} & \leq & E\left[\sum_{j=1}^{M}\int_{N}{\left|\hat{e}\left(y|\theta_{j}\right)\right|}^{2}q^{-1}\left(y\right)dV\right]\\ & = & E\left[\sum_{j=1}^{M}\int_{N}{\left(\sum_{k=K_{1}+1}^{\infty }c_{Y|\theta_{j},k}\psi_{k}\left(y\right)+\sum_{k=1}^{K_{1}}\left(c_{Y|\theta_{j},k}-{\hat{c}}_{Y|\theta_{j},k}\right)\psi_{k}\left(y\right)\right)}^{2}q\left(y\right)dV\right],\;\; \end{array}$$

where the first inequality follows from Jensen’s inequality. Here, the randomness comes from the estimators ${\hat{c}}_{Y|\theta_{j},k}$. Due to the orthonormality of basis functions, $\psi_{k}\in L^{2}\left(N,q\right)$, the error estimation in (27) can be simplified as,

(28) $$\begin{array}{lll} {\left(E\left[{\hat{e}}_{L^{2}}\right]\right)}^{2} & \leq & \sum_{j=1}^{M}\sum_{k=K_{1}+1}^{\infty }{\left[c_{Y|\theta_{j},k}\right]}^{2}+\sum_{j=1}^{M}\sum_{k=1}^{K_{1}}E_{Y|\theta_{j}}\left[{\left(c_{Y|\theta_{j},k}-{\hat{c}}_{Y|\theta_{j},k}\right)}^{2}\right],\\ & = & \sum_{j=1}^{M}\sum_{k=K_{1}+1}^{\infty }{\left[c_{Y|\theta_{j},k}\right]}^{2}+\frac{1}{N}\sum_{j=1}^{M}\sum_{k=1}^{K_{1}}{\mathrm{Var}}_{Y|\theta_{j}}\left[\psi_{k}\left(Y\right)\right], \end{array}$$

where the inequality follows from the linearity of expectation, and the equality follows from $E{\hat{c}}_{Y|\theta_{j},k}=c_{Y|\theta_{j},k}$ in (21) and Var$\left({\hat{c}}_{Y|\theta_{j},k}\right)=\frac{1}{N}{\mathrm{Var}}_{Y|\theta_{j}}\left[\psi_{k}\left(Y\right)\right]$ in (22). In error estimation (28), the first term is deterministic, and the second term is random. We have so far proven that the expectation $E\left[{\hat{e}}_{L^{2}}\right]$ is bounded by (25). Similarly, we can prove that the variance Var$\left[{\hat{e}}_{L^{2}}\right]$ is bounded by (26).

Next, we prove that the expectation $E\left[{\hat{e}}_{L^{2}}\right]$ converges to zero as $K_{1}\to \infty$ and then N→∞. Parseval’s theorem states that:

(29) $$\sum_{k=1}^{\infty }{\left[c_{Y|\theta_{j},k}\right]}^{2}=\int_{N}p{\left(y|\theta_{j}\right)}^{2}q^{-1}\left(y\right)dV<+\infty ,\;\;\mathrm{for}\;\mathrm{all}\;\theta_{j},$$

where the inequality follows from $p\left(y|\theta_{j}\right)\in H_{q^{-1}}\left(N\right)\subset L^{2}\left(N,q^{-1}\right)$ for all $\theta_{j}$. For ∀ε>0, there exists an integer ${\tilde{K}}_{1}\left(\theta_{j}\right)$ for $\theta_{j}$ such that:

(30) $$\sum_{k={\tilde{K}}_{1}\left(\theta_{j}\right)}^{\infty }{\left[c_{Y|\theta_{j},k}\right]}^{2}<\frac{\varepsilon }{2M}.$$

Let:

(31) $$K_{1}=\max\left\{{\tilde{K}}_{1}\left(\theta_{1}\right),\ldots ,{\tilde{K}}_{1}\left(\theta_{M}\right)\right\},$$

then the first term in (28) can be bounded by: ε/2,

(32) $$\sum_{j=1}^{M}\sum_{k=K_{1}+1}^{\infty }{\left[c_{Y|\theta_{j},k}\right]}^{2}<\frac{\varepsilon }{2}.$$

Since the variance Var${}_{Y|\theta_{j}}\left[\psi_{k}\left(Y\right)\right]$ is assumed to be finite for all *k* and *j*, there exists a constant D>0 such that Var${}_{Y|\theta_{j}}\left[\psi_{k}\left(Y\right)\right]$ can be bounded above by this constant *D*,

(33) $${\mathrm{Var}}_{Y|\theta_{j}}\left[\psi_{k}\left(Y\right)\right]\leq D,\;\;\;\mathrm{for}\;\mathrm{all}\;k=1,\ldots ,K_{1}\;\mathrm{and}\;j=1,\ldots ,M.$$

Then, for ∀ε>0, there exists a sufficiently large number of training data:

(34) $$N_{\min}=\frac{2MK_{1}D}{\varepsilon }.$$

such that whenever $N>N_{\min}$, then:

(35) $$\frac{1}{N}\sum_{j=1}^{M}\sum_{k=1}^{K_{1}}{\mathrm{Var}}_{Y|\theta_{j}}\left[\psi_{k}\left(Y\right)\right]<\frac{\varepsilon }{2}.$$

Since ε>0 is arbitrary, by substituting Equation (32) and Equation (35) into the error estimation (28), we obtain that $E\left[{\hat{e}}_{L^{2}}\right]$ converges to zero as $K_{1}\to \infty$ and then N→∞. Note that, we first take $K_{1}\to \infty$ to ensure the first error term in (28) vanishes and then take N→∞ to ensure the second error term in (28) vanishes. Thus, the limiting operations of $K_{1}$ and *N* are not commutative. Similarly, we can prove that the variance Var$\left[{\hat{e}}_{L^{2}}\right]$ converges to zero as $K_{1}\to \infty$ and then N→∞. □

Theorem 1 provides the intuition for specifying the number of training observation data *N* to achieve any desired accuracy ε>0 given fixed *M*-parameters and sufficiently large $K_{1}$. It can be seen from Theorem 1 that numerically, the expectation $E\left[{\hat{e}}_{L^{2}}\right]$ in (25) and the variance Var$\left[{\hat{e}}_{L^{2}}\right]$ in (26) can be bounded within arbitrarily small ε by choosing sufficiently large $K_{1}$ and *N*. Specifically, there are two error terms in Equations (25) and (26), the first being deterministic, induced by modes $k>K_{1}$, and the second random, induced by modes $k\leq K_{1}$. For the deterministic term ($k>K_{1}$), the error can be bounded by ε/2 by choosing sufficiently large $K_{1}$ satisfying (31). In our implementation, the number of basis functions $K_{1}$ is empirically chosen to be large enough in order to make the first error term in Equations (25) and (26) for $k>K_{1}$ as small as possible.

For the random term ($k\leq K_{1}$), the error can be bounded by ε/2 by choosing sufficiently large *N* satisfying $N>N_{\min}=2MK_{1}D/\varepsilon$ (Equation (34)). The minimum number of training data, $N_{\min}$, depends on the upper bound of Var${}_{Y|\theta_{j}}\left[\psi_{k}\left(Y\right)\right]$, *D*. However, the upper bound *D* may not exist for some problems. This means that for some problems, the assumption for finite Var${}_{Y|\theta_{j}}\left[\psi_{k}\left(Y\right)\right]$ in Theorem 1 may not be satisfied. Even if the upper bound *D* exists, it is typically not easy to evaluate its value given an arbitrary basis $\psi_{k}\in L^{2}\left(N,q\right)$ since one needs to evaluate Var${}_{Y|\theta_{j}}\left[\psi_{k}\left(Y\right)\right]$ for all $k=1,\ldots ,K_{1}$ and j=1,…,M. Note that Theorem 1 holds true for representing $\hat{p}\left(y|\theta_{j}\right)$ with an arbitrary basis $\left\{\psi_{k}\right\}\in L^{2}\left(N,q\right)$ as long as $p\left(y|\theta_{j}\right)\in H_{q^{-1}}\left(N\right)$ for all $\theta_{j}$ and Var${}_{Y|\theta_{j}}\left[\psi_{k}\left(Y\right)\right]$ is finite for $k\leq K_{1}$ and j=1,…,M. Next, we provide several cases in which Var${}_{Y|\theta_{j}}\left[\psi_{k}\left(Y\right)\right]$ is finite for *k* and *j*.

**Remark** **1.** *If the weighted Hilbert space $L^{2}\left(N,q\right)$ is defined on a compact manifold N and has smooth basis functions $\psi_{k}$, then Var${}_{Y|\theta_{j}}\left[\psi_{k}\left(Y\right)\right]$ is finite for a fixed $k\in N^{+}$ and j=1,…,M. This assertion follows from the fact that continuous functions on a compact manifold are bounded. The smoothness assumption is not unreasonable in many applications since the orthonormal basis functions are obtained as solutions of an eigenvalue problem of a self-adjoint second-order elliptic differential operator. Note that the bound here is not necessarily a uniform bound of $\psi_{k}\left(Y\right)$ for all $k\in N^{+}$ and j=1,…,M. As long as Var${}_{Y|\theta_{j}}\left[\psi_{k}\left(Y\right)\right]$ is finite for $k\leq K_{1}$ and j=1,…,M, the upper bound D is finite, and then, Theorem 1 holds.*

**Remark** **2.** *If the manifold N is a hyperrectangle in $R^{n}$ and the weight q is a uniform distribution on N, then Var${}_{Y|\theta_{j}}\left[\psi_{k}\left(Y\right)\right]$ is finite for a fixed $k\in N^{+}$ and j=1,…,M. This assertion is an immediate consequence of Remark 1.*

In Theorem 1, $N_{\min}$ depends on the upper bound of Var${}_{Y|\theta_{j}}\left[\psi_{k}\left(Y\right)\right]$, *D*, as shown in (34). In the following, we will specify a Hilbert space, referred to as a data-driven Hilbert space, so that $N_{\min}$ is independent of *D* and is only dependent of *M*, $K_{1}$, and ε. As a consequence, we can easily determine how many training data *N* for bounding the second error term in Equations (25) and (26).

### 3.2. Error Estimation Using a Data-Driven Hilbert Space

We now turn to the discussion of a specific data-driven Hilbert space $L^{2}\left(N,\bar{q}\right)$ with orthonormal basis functions ${\bar{\psi }}_{k}$. Our goal is to specify the weight function $\bar{q}$ such that the minimum number of training data, ${\bar{N}}_{\min}$, only depends on *M*, $K_{1}$, and ε. Here, the overline $\bar{\cdot }$ corresponds to the specific data-driven Hilbert space. The second error term in (28) can be further estimated as,

(36) $$\begin{array}{lll} \frac{1}{N}\sum_{j=1}^{M}\sum_{k=1}^{K_{1}}{\mathrm{Var}}_{Y|\theta_{j}}\left[{\bar{\psi }}_{k}\left(Y\right)\right] & \leq & \frac{1}{N}\sum_{k=1}^{K_{1}}\sum_{j=1}^{M}E_{Y|\theta_{j}}\left[{\bar{\psi }}_{k}^{2}\left(Y\right)\right]=\frac{M}{N}\sum_{k=1}^{K_{1}}\int_{N}{\bar{\psi }}_{k}^{2}\left(y\right)\left(\frac{1}{M}\sum_{j=1}^{M}p\left(y|\theta_{j}\right)\right)dV,\;\; \end{array}$$

where the basis functions are substituted with the specific ${\bar{\psi }}_{k}$. Notice that ${\bar{\psi }}_{k}\left(y\right)$ are orthonormal basis functions with respect to the weight $\bar{q}$ in $L^{2}\left(N,\bar{q}\right)$. One specific choice of the weight function $\bar{q}\left(y\right)$ is:

(37) $$\bar{q}\left(y\right)=\frac{1}{M}\sum_{j=1}^{M}p\left(y|\theta_{j}\right),$$

where $\bar{q}\left(y\right)$ has been normalized, i.e.,

(38) $$\int_{N}\bar{q}\left(y\right)dV=\int_{N}\frac{1}{M}\sum_{j=1}^{M}p\left(y|\theta_{j}\right)dV=1.$$

For the data-driven Hilbert space, we always use a normalized weight function $\bar{q}\left(y\right)$. Note that the weight function $\bar{q}\left(y\right)$ in (37) is a discretization of the marginal density function of Y with Θ marginalized out,

(39) $$\bar{q}\left(y\right)=\frac{1}{M}\sum_{j=1}^{M}p\left(y|\theta_{j}\right)\approx \int_{M}p\left(y|\theta \right)\tilde{q}\left(\theta \right)d\theta =\int_{M}p\left(y,\theta \right)d\theta ,$$

where p(y,θ) denotes the joint density of (Y,Θ). Essentially, the weight function $\bar{q}\left(y\right)$ in (37) is the sampling density of all the training data ${\left\{y_{i,j}\right\}}_{j=1,\ldots ,M}^{i=1,\ldots ,N}$, which motivates us to refer to $L^{2}\left(N,\bar{q}\right)$ as a data-driven Hilbert space.

Next, we prove that by specifying the data-driven basis functions ${\bar{\psi }}_{k}\in L^{2}\left(N,\bar{q}\right)$, the variance Var${}_{Y|\theta_{j}}\left[{\bar{\psi }}_{k}\left(Y\right)\right]$ is finite for all $k\in N^{+}$ and j=1,…,M. Subsequently, we can obtain the minimum number of training data, ${\bar{N}}_{\min}$, to only depend on *M*, $K_{1}$, and ε, such that the expectation $E\left[{\hat{e}}_{L^{2}}\right]$ in (25) and the variance Var$\left[{\hat{e}}_{L^{2}}\right]$ in (26) are bounded above by any ε>0.

**Proposition** **2.** *Let ${\left\{y_{i,j}\right\}}_{i=1,\ldots ,N}$ be i.i.d. samples of $Y|\theta_{j}$ with density $p(y|\theta_{j})$. Let $p\left(y|\theta_{j}\right)\in H_{q^{-1}}\left(N\right)$ for all ${\left\{\theta_{j}\right\}}_{j=1,\ldots ,M}$ with weight $\bar{q}$ specified in (37), and let $\left\{{\bar{\psi }}_{k}\right\}$ be the complete orthonormal basis of $L^{2}\left(N,\bar{q}\right)$. Then, Var${}_{Y|\theta_{j}}\left[{\bar{\psi }}_{k}\left(Y\right)\right]$ is finite for all $k\in N^{+}$ and j=1,…,M.*

**Proof.**  Notice that for all $k\in N^{+}$, we have:

(40) $$\begin{array}{lll} \frac{1}{M}\sum_{j=1}^{M}{\mathrm{Var}}_{Y|\theta_{j}}\left[{\bar{\psi }}_{k}\left(Y\right)\right] & \leq & \frac{1}{M}\sum_{j=1}^{M}E_{Y|\theta_{j}}\left[{\bar{\psi }}_{k}^{2}\left(Y\right)\right]=\int_{N}{\bar{\psi }}_{k}^{2}\left(y\right)\frac{1}{M}\sum_{j=1}^{M}p\left(y|\theta_{j}\right)dV\\ & = & \int_{N}{\bar{\psi }}_{k}^{2}\left(y\right)\bar{q}\left(y\right)dV=1, \end{array}$$

where the last equality follows directly from the orthonormality of basis functions ${\bar{\psi }}_{k}\left(y\right)\in L^{2}\left(N,\bar{q}\right)$. From Equation (40), we can obtain that for all $k\in N^{+}$ and j=1,…,M, the variance Var${}_{Y|\theta_{j}}\left[{\bar{\psi }}_{k}\left(Y\right)\right]$ is finite. □

**Theorem** **2.** *Given the same hypothesis as in Proposition 2, then:*

(41) $$\begin{array}{lll} E\left[{\hat{e}}_{L^{2}}\right] & \leq & {\left(\sum_{j=1}^{M}\sum_{k=K_{1}+1}^{\infty }{\left[c_{Y|\theta_{j},k}\right]}^{2}+\frac{MK_{1}}{N}\right)}^{\frac{1}{2}}, \end{array}$$

(42) $$\begin{array}{lll} Var\left[{\hat{e}}_{L^{2}}\right] & \leq & \sum_{j=1}^{M}\sum_{k=K_{1}+1}^{\infty }{\left[c_{Y|\theta_{j},k}\right]}^{2}+\frac{MK_{1}}{N}. \end{array}$$

*where ${\hat{e}}_{L^{2}}$ is defined by (24) and $c_{Y|\theta_{j},k}$ is given by (19). Moreover, $E\left[{\hat{e}}_{L^{2}}\right]$ and Var$\left[{\hat{e}}_{L^{2}}\right]$ converge to zero as $K_{1}\to \infty$ and then N→∞, where the limiting operations of $K_{1}$ and N are not commutative.*

**Proof.**  According to Proposition 2, we have that the variance Var${}_{Y|\theta_{j}}\left[{\bar{\psi }}_{k}\left(Y\right)\right]$ is finite for all $k\in N^{+}$ and j=1,…,M. According to Proposition 1, since Var${}_{Y|\theta_{j}}\left[{\bar{\psi }}_{k}\left(Y\right)\right]$ is finite, we have that the estimator ${\hat{c}}_{Y|\theta_{j},k}$ is both unbiased and consistent for $c_{Y|\theta_{j},k}$. All conditions in Theorem 1 are satisfied, so that we can obtain the error estimation of the expectation $E\left[{\hat{e}}_{L^{2}}\right]$ in (25) and the error estimation of the variance Var$\left[{\hat{e}}_{L^{2}}\right]$ in (26). Moreover, the second error term in $E\left[{\hat{e}}_{L^{2}}\right]$ (25) and Var$\left[{\hat{e}}_{L^{2}}\right]$ (26) can be both bounded by Equation (40), so that we can obtain our error estimations (41) and (42).

Choose $K_{1}$ as in (31) such that the first term in (41) and (42) is bounded by ε/2. The second term $MK_{1}/N$ in (41) and (42) can be bounded by an arbitrarily small ε/2 if the number of training data *N* satisfies:

(43) $$N>{\bar{N}}_{\min}\equiv 2MK_{1}/\varepsilon .$$

Then, both the expectation $E\left[{\hat{e}}_{L^{2}}\right]$ and the variance Var$\left[{\hat{e}}_{L^{2}}\right]$ can be bounded by ε. Since ε>0 is arbitrary, the proof is complete. □

Recall that by applying arbitrary basis functions to represent $\hat{p}\left(y|\theta \right)$ in (10), it is typically not easy to evaluate the upper bound *D* in (33), which implies that it is not easy to determine how many observation data, $N_{\min}$ (Equation (34)), should be used for training. However, by applying the data-driven basis functions ${\bar{\psi }}_{k}$ to represent $\hat{p}\left(y|\theta \right)$ in (10), the minimum number of training data, ${\bar{N}}_{\min}$ (Equation (43)), becomes independent of *D*, and is only dependent of *M*, $K_{1}$, and ε, as can be seen from Theorem 2. To let the error induced by modes *k*$\leq K_{1}$ be smaller than a desired ε/2, we can easily determine how many observation data, ${\bar{N}}_{\min}$ (Equation (43)), should be used for training. In this sense, the specific data-driven Hilbert space $L^{2}\left(N,\bar{q}\right)$ with the corresponding basis functions ${\bar{\psi }}_{k}$ is a good choice for representing (10).

We have so far theoretically verified the validity of the representation (10) in estimating the conditional density $p(y|\theta_{j})$ (Theorem 1). In particular, using the data-driven basis ${\bar{\psi }}_{k}\in L^{2}\left(N,\bar{q}\right)$, we can easily control the error of conditional density estimation by specifying the number of training data *N* (Theorem 2). To summarize, the training procedures can be outlined as follows:

(*1-A*) Generate the training dataset, including training parameters ${\left\{\theta_{j}\right\}}_{j=1,\ldots ,M}$ and observations ${\left\{y_{i,j}\right\}}_{j=1,\ldots ,M}^{i=1,\ldots ,N}$. The length of training data *N* is empirically determined based on the criteria (34) or (43).
(*1-B*) Construct the basis functions for parameter θ space and for observation y space by using the training dataset. For y space, we need to empirically choose the number of basis functions $K_{1}$ to let the error induced by modes $k>K_{1}$ be as small as possible. In particular, for the data-driven Hilbert space, we will provide a detailed discussion on how to estimate the data-driven basis functions of $L^{2}\left(N,\bar{q}\right)$ with the sampling density $\bar{q}$ from the training data in the following Section 4. Note that this basis estimation will introduce additional errors beyond the results in this section, which assumed the data-driven basis functions to be given.
(*1-C*) Train the matrix $C_{Y\Theta }$ in (12) and then estimate the conditional density $\hat{p}\left(y|\theta \right)$ by using the nonparametric RKWHS representation (10) with the expansion coefficients ${\hat{c}}_{Y|\theta ,k}$ (15).
(*1-D*) Finally, for new observations $y^{†}=\left\{y_{1}^{†},\ldots ,y_{T}^{†}\right\}$, define the likelihood function as a product of the conditional densities of new observations $y^{†}$ given any θ,
  (44) $$p\left(y^{†}|\theta \right)\equiv \prod_{t=1}^{T}\hat{p}\left(y_{t}^{†}|\theta \right).$$

Next, we address the second important question for the RKWHS representation (Procedure (*1-B*)): how to construct basis functions for θ and y. Especially, we focus on how to construct the data-driven basis functions for y.

## 4. Basis Functions

This section will be organized as follows. In Section 4.1, we discuss how to employ analytical basis functions for parameter θ and for observation y as in the usual polynomial chaos expansion. In Section 4.2, we discuss how to construct the data-driven basis functions ${\bar{\psi }}_{k}\in L^{2}\left(N,\bar{q}\right)$ with N being the manifold of the training dataset ${\left\{y_{i,j}\right\}}_{j=1,\ldots ,M}^{i=1,\ldots ,N}$ and the weight $\bar{q}$ by (37) being the sampling density of ${\left\{y_{i,j}\right\}}_{j=1,\ldots ,M}^{i=1,\ldots ,N}$.

### 4.1. Analytic Basis Functions

If no prior information about the parameter space other than its domain is known, we can assume that the training parameters are uniformly distributed on the parameter θ space. In particular, we choose *M* number of well-sampled training parameters ${\left\{\theta_{j}\right\}}_{j=1,\ldots ,M}={\left\{\theta_{j}^{1},\ldots ,\theta_{j}^{m}\right\}}_{j=1,\ldots ,M}$ in an *m*-dimensional box $M\subset R^{m}$,

(45) $$M\equiv \left[\theta_{\min}^{1},\theta_{\max}^{1}\right]\times \cdots \times \left[\theta_{\min}^{m},\theta_{\max}^{m}\right],$$

where × denotes a Cartesian product and the two parameters $\theta_{\min}^{s}$ and $\theta_{\max}^{s}$ are the minimum and maximum values of the uniform distribution for the $s^{\mathrm{th}}$ coordinate of θ space. Here, the well-sampled uniform distribution corresponds to a regular grid, which is a tessellation of *m*-dimensional Euclidean space $R^{m}$ by congruent parallelotopes. Two parameters $\theta_{\min}^{s}$ and $\theta_{\max}^{s}$ are determined by:

(46) $$\begin{array}{lll} \theta_{\min}^{s} & = & \min_{j=1,\ldots ,M}\left\{\theta_{j}^{s}\right\}-\gamma \left(\max_{j=1,\ldots ,M}\left\{\theta_{j}^{s}\right\}-\min_{j=1,\ldots ,M}\left\{\theta_{j}^{s}\right\}\right),\\ \theta_{\max}^{s} & = & \max_{j=1,\ldots ,M}\left\{\theta_{j}^{s}\right\}+\gamma \left(\max_{j=1,\ldots ,M}\left\{\theta_{j}^{s}\right\}-\min_{j=1,\ldots ,M}\left\{\theta_{j}^{s}\right\}\right). \end{array}$$

**Figure 1:**
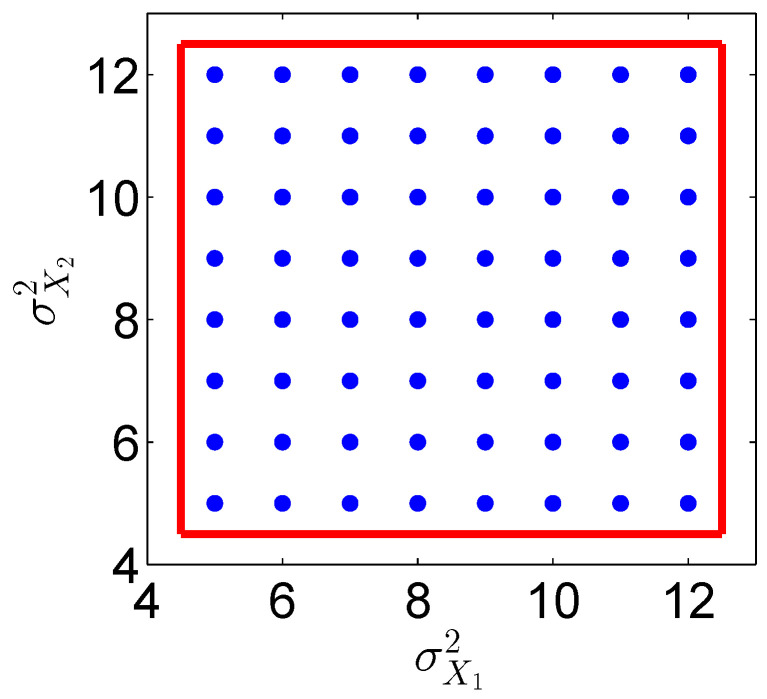
(Color online) An example of well-sampled 2D uniformly-distributed data points (blue circles). The boundary of the uniform distribution is depicted with a red square. Furthermore, these well-sampled data points correspond to the training parameters in Example I in Section 5. In this example, the well-sampled uniformly-distributed training parameters are $\left(\sigma_{X_{1}}^{2},\sigma_{X_{2}}^{2}\right)\in {\left\{(i,j)\right\}}_{i=5,\ldots ,12}^{j=5,\ldots ,12}$ (blue circles). The equal spacing distances of both coordinates are one. The two-dimensional box M is ${\left[4.5,12.5\right]}^{2}$ (red square).

For *M* regularly-spaced grid points $\theta_{j}^{s}$, we set $\gamma =.5{\left(M^{s}-1\right)}^{-1}$ in all of our numerical examples below, where $M^{s}$ is the number of training parameters in the $s^{\mathrm{th}}$ coordinate. For example, see Figure 1 for the 2D well-sampled uniformly-distributed data {(5,5),(6,5),…,(12,12)} (blue circles). In this case, the two-dimensional box M is ${\left[4.5,12.5\right]}^{2}$ (red square).

On this simple geometry, we will choose $\varphi_{k}$ to be the tensor product of the basis functions on each coordinate. Notice that we have taken the weight function $\tilde{q}$ to be the sampling density of the training parameters in order to simplify the expansion coefficient ${\hat{c}}_{Y|\theta ,k}$ in (15). In this case, the weight $\tilde{q}$ is a uniform distribution on M. Then, for the $s^{\mathrm{th}}$ coordinate of the parameter, $\theta^{s}$, the weight function ${\tilde{q}}^{s}\left(\theta^{s}\right)$ is a uniform distribution on the interval $\left[\theta_{\min}^{s},\theta_{\max}^{s}\right]$, and one can choose the following cosine basis functions,

(47) $$\Phi_{k^{s}}\left(\theta^{s}\right)=\left\{\begin{array}{c} 1,\;\;\;\;\;\;\;\;\;\;\;\;\;\;\;\;\;\;\;\;\;\mathrm{if}\;k^{s}=0,\\ \sqrt{2}\cos\left(k^{s}\pi \frac{\theta^{s}-\theta_{\min}^{s}}{\theta_{\max}^{s}-\theta_{\min}^{s}}\right),\;\mathrm{else}, \end{array}\right.$$

where $\Phi_{k^{s}}\left(\theta^{s}\right)$ form a complete orthonormal basis of $L^{2}\left(\left[\theta_{\min}^{s},\theta_{\max}^{s}\right],{\tilde{q}}^{s}\right)$. This choice of basis functions corresponds to exactly the data-driven basis functions produced by the diffusion maps algorithm on the uniformly-distributed dataset on a compact interval, which will be discussed in Section 4.2. Although other choices such as the Legendre polynomials can be used, this choice will lead to a larger value of constant *D* in (34) that controls the minimum number of training data for accurate estimation.

Subsequently, we set $L^{2}\left(M,\tilde{q}\right)={⨂}_{s=1}^{m}L^{2}\left(\left[\theta_{\min}^{s},\theta_{\max}^{s}\right],{\tilde{q}}^{s}\right)$, where ⊗ denotes the Hilbert tensor product, and $\tilde{q}\left(\theta \right)={⨂}_{s=1}^{m}{\tilde{q}}^{s}\left(\theta^{s}\right)$ is the uniform distribution on the *m*-dimensional box M. Correspondingly, the basis functions $\varphi_{k}\left(\theta \right)$ are a tensor product of $\Phi_{k^{s}}\left(\theta^{s}\right)$ for s=1,…,m,

(48) $$\varphi_{k}\left(\theta \right)=\overset{m}{\underset{s=1}{⨂}}\Phi_{k^{s}}\left(\theta^{s}\right)=\Phi_{k^{1}}\left(\theta^{1}\right)⊗\ldots ⊗\Phi_{k^{m}}\left(\theta^{m}\right),$$

where $k=\left(k^{1},\ldots ,k^{m}\right)$ and $\theta =\left(\theta^{1},\ldots ,\theta^{m}\right)$. Based on the property of the tensor product of Hilbert spaces, $\left\{\varphi_{k}\left(\theta \right)\right\}$ forms a complete orthonormal basis of $L^{2}\left(M,\tilde{q}\right)$.

We now turn to the discussion of how to construct analytic basis functions for y. The approach is similar to the one for parameter θ, except that the domain of the data is specified empirically and the weight function is chosen to correspond to some well-known analytical basis functions, independent of the sampling distribution of the data y. That is, we assume the geometry of the data has the following tensor structure, $N=N^{1}\times \ldots \times N^{n}$, where $N^{s}$ will be specified empirically based on the ambient space coordinate of y. Let $y^{s}$ be the $s^{\mathrm{th}}$ ambient component of y; we can choose a weighted Hilbert space $L^{2}\left(N^{s},q^{s}\left(y^{s};\alpha^{s}\right)\right)$ with the weight $q^{s}$ depending on the parameters $\alpha^{s}$ and being normalized to satisfy $\int_{R}q^{s}\left(y^{s};\alpha^{s}\right)dy^{s}=1$. For each coordinate, let $\Psi_{k^{s}}\left(y^{s};\alpha^{s}\right)$ be the corresponding orthonormal basis functions, which possess analytic expressions. Subsequently, we can obtain a set of complete orthonormal basis functions $\psi_{k}\in L^{2}\left(N,q\right)$ for y by taking the tensor product of these $\Psi_{k^{s}}$ as in (48).

For example, if the weight $q^{s}$ is uniform, $N^{s}\subset R$ is simply a one-dimensional interval. In this case, we can choose the cosine basis functions $\Psi_{k^{s}}$ for y as in (47) such that the parameters $\alpha^{s}$ correspond to the boundaries of the domain $N^{s}$, which can be estimated as in (46). In our numerical experiments below, we will set γ=0.1. Another choice is to set the weight $q^{s}\left(y^{s};\alpha^{s}\right)$ to be Gaussian. In this case, the domain is assumed to be the real line, $N^{s}=R$. For this choice, the corresponding orthonormal basis functions $\Psi_{k^{s}}$ are Hermite polynomials, and the parameters $\alpha^{s}$, corresponding to the mean and variance of the Gaussian distribution, can be empirically estimated from the training data.

In the remainder of this paper, we will always use the cosine basis functions for θ. The application of (10) using cosine basis functions for y is referred to as the cosine representation. The application of (10) using Hermite basis functions for y is referred to as the Hermite representation.

### 4.2. Data-Driven Basis Functions

In this section, we discuss how to construct a set of data-driven basis functions ${\bar{\psi }}_{k}$∈$L^{2}\left(N,\bar{q}\right)$ with N being the manifold of the training dataset ${\left\{y_{i,j}\right\}}_{j=1,\ldots ,M}^{i=1,\ldots ,N}$ and weight $\bar{q}$ in (37) being the sampling density of $\left\{y_{i,j}\right\}$ for all i=1,…,N, and j=1,…,M. The issues here are that the analytical expression of the sampling density $\bar{q}$ is unknown and the Riemannian metric inherited by the data manifold N from the ambient space $R^{n}$ is also unknown. Fortunately, these issues can be overcome by the diffusion maps algorithm [18,19,20].

#### 4.2.1. Learning the Data-Driven Basis Functions

Given a dataset $y_{i,j}\in N\subseteq R^{n}$ with the sampling density $\bar{q}\left(y\right)$ (37), defined with respect to the volume form inherited by the manifold N from the ambient space $R^{n}$, one can use the kernel-based diffusion maps algorithm to construct an MN×MN matrix L that approximates a weighted Laplacian operator, $L=\nabla \log\left(\bar{q}\right)\cdot \nabla +△$, that takes functions with Neumann boundary conditions for the compact manifold N with the boundary if the manifold has a boundary. The eigenvectors ${\bar{\psi }}_{k}$ of the matrix L are discrete approximations of the eigenfunctions ${\bar{\psi }}_{k}\left(y\right)$ of the operator L, which form an orthonormal basis of the weighted Hilbert space $L^{2}\left(N,\bar{q}\right)$. Connecting to the discussion on the RKWHS in Section 2, the eigenfunctions of $L^{*}=-div\left(\nabla \log\bar{q}\;\;\right)+\Delta$, that is $\left\{\Psi_{k}:={\bar{\psi }}_{k}\bar{q}\right\}$, can be approximated using an integral operator in (7) with the appropriate kernel constructed by the diffusion maps algorithm, up to a diagonal conjugation. Basically, $H_{{\bar{q}}^{-1}}\left(N\right)$ is the data-driven reproducing kernel Hilbert space defined with the feature map in (9), induced by eigenfunctions of $L^{*}$.

Each component of the eigenvector ${\bar{\psi }}_{k}\in R^{MN}$ is a discrete estimate of the eigenfunction ${\bar{\psi }}_{k}\left(y_{i,j}\right)$, evaluated at the training data point $y_{i,j}$. The sampling density $\bar{q}$ defined in (37) is estimated using a kernel density estimation method [22]. In contrast to the analytic continuous basis functions in the above Section 4.1, the data-driven basis functions ${\bar{\psi }}_{k}\in L^{2}\left(N,\bar{q}\right)$ are represented nonparametrically by the discrete eigenvectors ${\bar{\psi }}_{k}\in R^{MN}$ using the diffusion maps algorithm. The outcome of the training is a discrete estimate of the conditional density, $\hat{p}\left(y_{i,j}|\theta \right)$, which estimates the representation $\hat{p}\left(y|\theta \right)$ (10) on each training data point $y_{i,j}$.

In our implementation, we use the Variable-Bandwidth Diffusion Maps (VBDM) algorithm introduced in [20], which extends the diffusion maps to non-compact manifolds without a boundary. See the supplementary material of [23] for the MATLAB code of this algorithm. We should point out that this discrete approximation induces errors in the basis function, which are estimated in detail in [24]. These errors are in addition to the error estimations in Section 3.

We note that if the data are uniformly distributed on a one-dimensional bounded interval, then the VBDM solutions are the cosine basis functions, which are eigenfunctions of the Laplacian operator on bounded interval with Neumann boundary conditions. This means that the cosine functions in (47) that are used to represent each component of θ are analogous to the data-driven basis functions. The difference is that with the parametric choice in (47), one avoids VBDM at the expense of specifying the boundaries of the domain, $\left[\theta_{\min}^{s},\theta_{\max}^{s}\right]$. In the remainder of this paper, we refer to an application of (10) with cosine basis functions for θ and VBDM basis functions for y as the VBDM representation.

However, a direct application of the VBDM algorithm suffers from the expensive computational cost for large training data. Basically, we need an algorithm that allows us to subsample from the training dataset while preserving the sampling distribution of the full dataset. In Appendix A, we provide a simple box-averaging method to achieve this goal. In the remainder of this paper, we will denote the reduced data obtained via the box-averaging method in Appendix A by ${\left\{{\bar{y}}_{b}\right\}}_{b=1,\ldots ,B}$, where B≪MN. We refer to them as the box-averaged data points. When the number of training data is too large, we apply the VBDM algorithm on these box-averaged data to obtain the discrete estimate of the eigenfunctions ${\bar{\psi }}_{k}\left({\bar{y}}_{b}\right)$.

The second issue arises from the discrete representation of the conditional density in the observation y space using the VBDM algorithm. Notice that the VBDM representation, $\hat{p}\left(y_{i,j}|\theta \right)$, is only estimated at each training data point $y_{i,j}$. A natural problem is to extend the representation onto new observations $y_{t}\notin {\left\{y_{i,j}\right\}}_{j=1,\ldots ,M}^{i=1,\ldots ,N}$ that are not part of the training dataset (Procedure (*1-D*)). Next, we address this issue.

#### 4.2.2. Nyström Extension

We now discuss an extension method to evaluate basis functions ${\bar{\psi }}_{k}$ on a new data point that does not belong to the training dataset. Given such an extension method, we can proceed with Procedure (*1-D*) by evaluating ${\bar{\psi }}_{k}\left(y_{t}\right)$ on new observations $y_{t}\notin {\left\{y_{i,j}\right\}}_{j=1,\ldots ,M}^{i=1,\ldots ,N}$, which in turn give $\hat{p}\left(y_{t}|\theta \right)$. Second, this extension is also needed in the training Procedure (*1-C*) when MN is large. More specifically, for training the matrix $C_{Y\Theta }$ in (12), we need to know the estimate of the eigenfunction ${\bar{\psi }}_{k}\left(y_{i,j}\right)$ for all the original training data $y_{i,j}$. Computationally, however, we can only construct the discrete estimate of the eigenfunction ${\bar{\psi }}_{k}\left({\bar{y}}_{b}\right)$ at the reduced box-averaged data points ${\bar{y}}_{b}$. This suggests that we need to extend the eigenfunctions ${\bar{\psi }}_{k}\left({\bar{y}}_{b}\right)$ onto all the original training data ${\left\{y_{i,j}\right\}}_{j=1,\ldots ,M}^{i=1,\ldots ,N}$.

For the convenience of discussion, the training data that are used to construct the eigenfunctions are denoted by ${\left\{y_{r}^{\mathrm{old}}\right\}}_{r=1,\ldots ,R}$, and all the data that are not part of ${\left\{y_{r}^{\mathrm{old}}\right\}}_{r=1,\ldots ,R}$ are denoted by $y^{\mathrm{new}}$. To extend the eigenfunctions ${\bar{\psi }}_{k}\left(y_{r}^{\mathrm{old}}\right)$ onto the data point $y^{\mathrm{new}}\notin {\left\{y_{r}^{\mathrm{old}}\right\}}_{r=1,\ldots ,R}$, one approach would be to use the Nyström extension [25] that is based on the basic theory of RKHS [26]. Let $H_{\bar{q}}\left(N\right)$ be the RKWHS with a symmetric positive kernel $\hat{T}:N\times N\to R$ defined as,

$$\begin{array}{l} \hat{T}\left(y,y^{\prime }\right)=\sum_{k=1}^{\infty }\lambda_{k}{\bar{\psi }}_{k}\left(y\right){\bar{\psi }}_{k}\left(y^{\prime }\right), \end{array}$$

where $\lambda_{k}$ is the corresponding eigenvalue of L associated with eigenfunction ${\bar{\psi }}_{k}$. Then, for any function $f\in H_{\bar{q}}\left(N\right)$, the Moore–Aronszajn theorem states that one can evaluate *f* at a∈N with the following inner product, $f\left(a\right)={\langle f,\hat{T}\left(a,\cdot \right)\rangle }_{H_{\bar{q}}}$. In our application, this amounts to evaluating,

(49) $${\bar{\psi }}_{k}\left(y^{\mathrm{new}}\right)=\frac{1}{R}\sum_{r=1}^{R}T\left(y^{\mathrm{new}},y_{r}^{\mathrm{old}}\right){\bar{\psi }}_{k}\left(y_{r}^{\mathrm{old}}\right),$$

where the non-symmetric kernel function T:N×N→R (constructed by the diffusion maps algorithm) is related to the symmetric kernel $\hat{T}$ by,

$$\begin{array}{l} T\left(y_{i},y_{j}\right)={\bar{q}}^{-1/2}\left(y_{i}\right)\hat{T}\left(y_{i},y_{j}\right){\bar{q}}^{1/2}\left(y_{j}\right) \end{array}$$

with $\bar{q}\left(y_{i}\right)$ being the sampling density of ${\left\{y_{r}^{\mathrm{old}}\right\}}_{r=1,\ldots ,R}$ at $y_{i}$. See the detailed evaluation of the kernels $\hat{T}$ and T for the Nyström extension in [27]. After obtaining the estimate of the eigenfunction ${\bar{\psi }}_{k}\left(y^{\mathrm{new}}\right)$ using the Nyström extension, we can train the matrix $C_{Y\Theta }$ in (12) for large MN and then obtain the representation of the conditional density on arbitrary new observation $y_{t}$, $\hat{p}\left(y_{t}|\theta \right)$.

To summarize this section, we have constructed two different sets of basis functions for y, the analytic basis functions of $L^{2}\left(N,q\right)$ such as the Hermite and cosine basis functions, which assume that the manifold is $R^{n}$ or hyperrectangle, respectively, and the data-driven basis functions of $L^{2}\left(N,\bar{q}\right),$ with N being the data manifold and $\bar{q}$ being the sampling density that are computed using the VBDM algorithm.

## 5. Parameter Estimation Using the Metropolis Scheme

First, we briefly review the Metropolis scheme for estimating the posterior density $p(\theta |y^{†})$ given new observations $y^{†}=\left\{y_{1}^{†},\ldots ,y_{T}^{†}\right\}$ for a specific parameter $\theta^{†}$. The key idea of the Metropolis scheme is to construct a Markov chain such that it converges to samples of conditional density $p(\theta |y^{†})$ as the target density. In our application, the parameter estimation procedures can be outlined as follows:

(*2-A*) Suppose we have $\theta_{0}∼p(\theta_{0}|y^{†})>0$, then for i≥1, we can sample $\theta^{*}∼\kappa \left(\theta_{i-1},\theta^{*}\right)$. Here, κ is the proposal kernel density. For example, use the random walk Metropolis algorithm to generate proposals, $\kappa \left(\theta_{i-1},\theta^{*}\right)=N\left(\theta_{i-1},C\right)$, where C, the proposal covariance, and is a tunable nuisance parameter.
(*2-B*) Accept the proposal, $\theta_{i}=\theta^{*}$ with probability $\min(\frac{p(\theta^{*}|y^{†})}{p(\theta_{i-1}|y^{†})},1)$, otherwise set $\theta_{i}=\theta_{i-1}$. Repeat Procedures (*2-A*) and (*2-B*) above. Notice that the posterior $p(\theta |y^{†})$ can be determined from the prior $p_{0}\left(\theta \right)$ and the likelihood $p(y^{†}|\theta )$ based on Bayes’ theorem (3). The likelihood function $p(y^{†}|\theta )$ is defined as a product of conditional densities of new observations $y^{†}=\left\{y_{1}^{†},\ldots ,y_{T}^{†}\right\}$ in (44) (Procedure (*1-D*)). The conditional densities of new observations $y^{†}$ given θ are obtained from the training Procedure (*1-C*).
(*2-C*) Generate a sufficiently long chain and use the chain’s statistic as an estimator of the true parameter $\theta^{†}$. Take multiple runs of the chain started at different initial $\theta_{0}$, and examine whether all these runs converge to the same distribution. The convergence of all the examples below has been validated using 10 randomly-chosen different initial conditions.

In the remainder of this section, we present numerical results of the Metropolis scheme using the proposed data-driven likelihood function on various instructive examples, where the likelihood function is either explicitly known, or can be approximated as in (6), or is intractable. In an example where the explicit likelihood is known, our goal is to show that the approach numerically converges to the true posterior estimate. In the second example, where the dimension of the data manifold is strictly less than the ambient dimension, we will show that the RKHS framework with the knowledge of the intrinsic geometry is superior. When the intrinsic geometrical information is unknown, the proposed data-driven likelihood function is competitive. In the third example with a low-dimensional dynamic and observation model of the form (5), we compare the proposed approach with standard methods, including the direct MCMC and nonintrusive spectral projection (both use the likelihood function of the form (6)). In our last example, we consider an observation model where the likelihood function is intractable and the cost of evaluating the observation model in (4) is numerically expensive.

### 5.1. Example I: Two-Dimensional Ornstein–Uhlenbeck Process

Consider an Ornstein–Uhlenbeck (OU) process as follows:

(50) $$dX=-\frac{1}{2}Xdt+\Sigma^{1/2}dW_{t},$$

where $X\equiv {\left(X_{1},X_{2}\right)}^{⊤}$ denotes the state variable, $W_{t}={\left(W_{1},W_{2}\right)}^{⊤}$ denotes two-dimensional Wiener processes, and $\Sigma \in R^{2\times 2}$ is a diagonal matrix with main diagonal components $\sigma_{X_{1}}^{2}$ and $\sigma_{X_{s}}^{2}$ to be estimated. In the stationary process, the solution of Equation (50) $X={\left(X_{1},X_{2}\right)}^{⊤}$ admits a Gaussian distribution $X∼N\left(0,\Sigma \right)$,

(51) $$p\left(X|\Sigma \right)=\det{\left(2\pi \Sigma \right)}^{-\frac{1}{2}}\exp\left[-\frac{1}{2}X^{⊤}\Sigma^{-1}X\right].$$

Our goal here is to estimate the posterior density and the posterior mean of the parameters $\left(\sigma_{X_{1}}^{2},\sigma_{X_{2}}^{2}\right)$, given a finite number, *T*, of observations, $X^{†}\equiv \left(X^{1†},\ldots ,X^{T†}\right)$, for hidden true parameters $\left({\left(\sigma_{X_{1}}^{2}\right)}^{†},{\left(\sigma_{X_{2}}^{2}\right)}^{†}\right)=\left(6.5,6.3\right)$, where each $X^{t†}$ is an i.i.d. sample of (51) for $\Sigma =\Sigma^{†}$. This example is shown here to verify the validity of the framework of our RKWHS representations for parameter estimation application.

One can show that the likelihood function for this problem is the inverse matrix gamma distribution, $\Sigma ∼IMG\left(\frac{T}{2}-\frac{3}{2},2,\Psi \right)$, where $\Psi =X^{†}{\left(X^{†}\right)}^{⊤}\in R^{2\times 2}$. If a prior is defined to be also the inverse matrix gamma distribution, $\Sigma ∼IMG\left(\alpha_{0},2,0\right)$, for some value of $\alpha_{0}$, then the posterior density $p(\Sigma |X^{†})$ can be obtained by applying Bayes’ theorem,

(52) $$p\left(\Sigma |X^{†}\right)∼IMG\left(\alpha_{0}+\frac{T}{2},2,\Psi \right).$$

The posterior mean can thereafter be obtained as,

(53) $${\left(\Sigma \right)}_{PM}=\left(\begin{array}{cc} {\left(\sigma_{X_{1}}^{2}\right)}_{PM} & 0\\ 0 & {\left(\sigma_{X_{2}}^{2}\right)}_{PM} \end{array}\right)=\frac{\Psi }{T+2\alpha_{0}-3}.$$

To compare with the analytic conditional density p(X|Σ) (51), we trained three RKWHS representations of the conditional density function, $\hat{p}\left(X|\Sigma \right)$, by using the same training dataset. For training, we used M=64 well-sampled uniformly-distributed training parameters (shown in Figure 1), $\left(\sigma_{X_{1}}^{2},\sigma_{X_{2}}^{2}\right)$, where $\sigma_{X_{j}}^{2}\in \left\{5,6,\ldots ,12\right\}$, which are denoted by ${\left\{\Sigma_{j}\right\}}_{j=1}^{M}$. For each training parameter $\Sigma_{j}$, we generated N= 640,000 well-sampled normally distributed observation data of density in (51) with $\Sigma =\Sigma_{j}$. For Hermite and cosine representations, we used 20 basis functions for each coordinate, and then, we could construct $K_{1}=400$ basis functions of two-dimensional observation, X, by taking the tensor product. For the VBDM representation, we first reduced the data from MN=8× 640,000 to $B=B^{1}\times B^{2}=100\times 100$ by the box-averaging method (Appendix A). Subsequently, we trained $K_{1}=400$ data-driven basis functions from the *B* box-averaged data using the VBDM algorithm [20].

**Figure 2:**
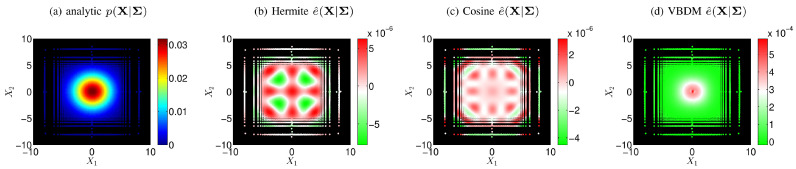
(Color online) (**a**) The analytic conditional density p(X|Σ) (51). For comparison, plotted are the pointwise errors of conditional density functions $\hat{e}\left(X|\Sigma \right)\equiv p\left(X|\Sigma \right)-\hat{p}\left(X|\Sigma \right)$ for (**b**) Hermite, (**c**) cosine, and (**d**) VBDM representations. The density and all the error functions are plotted on the B= 10,000 box-averaged data points. The training parameter $\Sigma \equiv \left(\sigma_{X_{1}}^{2},\sigma_{X_{2}}^{2}\right)=\left(5,5\right)$.

Figure 2a displays the analytic conditional density (51), and Figure 2b–d display the pointwise errors of the conditional densities $\hat{e}\left(X|\Sigma \right)\equiv p\left(X|\Sigma \right)-\hat{p}\left(X|\Sigma \right)$ for the training parameter $\left(\sigma_{X_{1}}^{2},\sigma_{X_{2}}^{2}\right)=\left(5,5\right)$. It can be seen from Figure 2b–d that all the pointwise errors are small compared to the analytic p(X|Σ) in Figure 2a, so that all representations of conditional densities $\hat{p}\left(X|\Sigma \right)$ are in excellent agreement with the analytic p(X|Σ) (Figure 2a). This suggests that for the Hermite representation, the upper bound *D* (33) in Theorem 1 is finite so that the representation is valid in estimating the conditional density, as can be seen from Figure 2b. On the other hand, the upper bounds *D* (33) for the cosine and the VBDM representations are always finite, as mentioned in Remark 2 and Proposition 2, respectively. We should also point out that for this example, the VBDM representation performed the worst with errors of order $10^{-4}$ compared to the Hermite and cosine representations whose errors were on the order of $10^{-6}$. This larger error in the VBDM representation was because the data-driven basis functions were estimated by discrete eigenvectors ${\bar{\psi }}_{k}\in R^{B}$, so additional errors [20] were introduced through this discrete approximation (especially on the high modes) on the box-averaged data, ${\left\{{\bar{y}}_{b}\right\}}_{b=1,\ldots ,B}$, B= 10,000. On the other hand, for Hermite and cosine representations, their analytic basis functions are known, so that the errors could be approximated by (25) in Theorem 1.

We now estimate the posterior density (52) and mean (53) by using the MCMC method (Procedures (*2-A*)–(*2-C*)). We generated T=400 well-sampled normally-distributed data as the observations from the true values of variance $\Sigma^{†}=\left({\left(\sigma_{X_{1}}^{2}\right)}^{†},{\left(\sigma_{X_{2}}^{2}\right)}^{†}\right)=\left(6.5,6.3\right)$. From the analytical Formula (53), we obtained the posterior mean as ${\left(\sigma_{X_{1}}^{2},\sigma_{X_{2}}^{2}\right)}_{PM}=\left(6.03,5.84\right)$. Here, the posterior mean deviated greatly from the true value since we only used T=400 normally-distributed observation data as new observations. If using much more new observation data, the analytical posterior mean (53) will get closer to the true value, $\left({\left(\sigma_{X_{1}}^{2}\right)}^{†},{\left(\sigma_{X_{2}}^{2}\right)}^{†}\right)$. In our simulation, we set the parameter in the prior, $\alpha_{0}=1$, and the proposal covariance, C=0.01I. For each chain, the initial condition $\left(\sigma_{X_{1},0}^{2},\sigma_{X_{2},0}^{2}\right)$ was drawn randomly from $U{\left[5,12\right]}^{2}$, and 800,000 iterations are generated for the chain.

**Figure 3:**
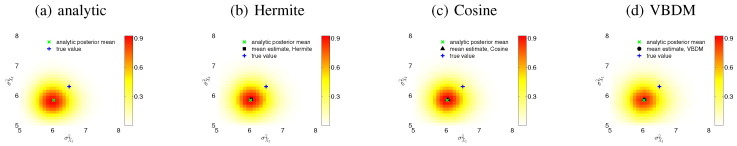
(Color online) Comparison of the posterior density functions $p(\Sigma |X^{†})$. (**a**) Analytical posterior density $p(\Sigma |X^{†})$ (52). (**b**) Hermite representation. (**c**) Cosine representation. (**d**) VBDM representation. The true value $\Sigma^{†}\equiv \left(\sigma_{X_{1}}^{2},\sigma_{X_{2}}^{2}\right)=\left(6.5,6.3\right)$ (blue plus). The analytic posterior mean is ${\left(\sigma_{X_{1}}^{2},\sigma_{X_{2}}^{2}\right)}_{PM}=\left(6.03,5.84\right)$ (green cross). The MCMC mean estimate using Hermite representation is $\left(\sigma_{X_{1}}^{2},\sigma_{X_{2}}^{2}\right)=\left(6.05,5.87\right)$ (black square). The MCMC mean estimate using Cosine representation is $\left(\sigma_{X_{1}}^{2},\sigma_{X_{2}}^{2}\right)=\left(6.05,5.87\right)$ (black triangle). The MCMC mean estimate using VBDM representation is $\left(\sigma_{X_{1}}^{2},\sigma_{X_{2}}^{2}\right)=\left(6.04,5.86\right)$ (black circle).

Figure 3b,c,d display the densities of the chain by using Hermite, cosine, and VBDM representation, respectively. The densities are plotted using the kernel density estimate on the chain ignoring the first 10,000 iterations. For comparison, Figure 3a displays the analytic posterior density (52). It can be seen from Figure 3 that the posterior densities by the three representations were in excellent agreement with each other and with the analytic posterior density (52). Figure 3 also shows the comparison between the posterior mean (53) and the MCMC mean estimates. From our numerical results, MCMC mean estimates by all representations and the analytic posterior mean (53) were identical within numerical accuracy. Therefore, for this 2D OU-process example, all representations were valid in estimating the posterior density and posterior mean of parameter Σ.

Next, we will investigate a system for which the intrinsic dimension *d* of the data manifold where the observations lie is smaller than the dimension of ambient space *n*.

### 5.2. Example II: Three-Dimensional System of SDE’s on a Torus

Consider a system of SDE’s on a torus defined in the intrinsic coordinates $\left(\theta ,\phi \right)\in {\left[0,2\pi \right)}^{2}$:

(54) $$d\left(\begin{array}{c} \theta \\ \phi \end{array}\right)=a\left(\theta ,\phi \right)dt+b\left(\theta ,\phi \right)\left(\begin{array}{c} dW_{1}\\ dW_{2} \end{array}\right),$$

where $W_{1}$ and $W_{2}$ are two independent Wiener processes, and the drift and diffusion coefficients are:

$$\begin{array}{lll} a\left(\theta ,\phi \right) & = & \left(\begin{array}{c} \frac{1}{2}+\frac{1}{8}\cos\left(\theta \right)\cos\left(2\phi \right)+\frac{1}{2}\cos\left(\theta +\pi /2\right)\\ 10+\frac{1}{2}\cos\left(\theta +\phi /2\right)+\cos\left(\theta +\pi /2\right) \end{array}\right),\\ b\left(\theta ,\phi \right) & = & \left(\begin{array}{cc} D+D\sin\left(\theta \right) & \frac{1}{4}\cos\left(\theta +\phi \right)\\ \frac{1}{4}\cos\left(\theta +\phi \right) & \frac{1}{40}+\frac{1}{40}\sin\left(\phi \right)\cos\left(\theta \right) \end{array}\right). \end{array}$$

The initial condition is $\left(\theta ,\phi \right)=\left(\pi ,\pi \right)$. Here, *D* is a parameter to be estimated. This example exhibits non-gradient drift, anisotropic diffusion, and multiple time scales. Both the observations and the training dataset were generated by numerically solving the SDE on appropriate parameters *D* in (54) with a time step Δt=0.1 and then mapping this data into the ambient space, $R^{3}$, via the standard embedding of the torus given by:

(55) $$x\equiv \left(x,y,z\right)=\left(\left(2+\sin\left(\theta \right)\right)\cos\left(\phi \right),\left(2+\sin\left(\theta \right)\right)\sin\left(\phi \right),\cos\left(\theta \right)\right).$$

Here, $x\equiv \left(x,y,z\right)$ are observations. This system on a torus satisfies d<n, where d=2 is the intrinsic dimension of x and n=3 is the dimension of ambient space $R^{n}$. Our goal is to estimate the posterior density and the posterior mean of parameter *D* given discrete-time observations of $x^{†}$, which are the solutions of (54) for a specific parameter $D^{†}$.

For training, we used M=8 well-sampled uniformly-distributed training parameters, ${\left\{D_{j}=j/4\right\}}_{j=1}^{8}$. For each training parameter $D_{j}$, we generated N= 54,000 observations of x by solving the SDE’s in (54) for parameter $D_{j}$. For Hermite and cosine representation, we constructed 10 basis functions for each x,y,z coordinate in Euclidean space. After taking tensor product of these basis functions, we could obtain $K_{1}=1000$ basis functions on the ambient space $R^{3}$. For VBDM representation, we first computed $B=B_{1}\times B_{2}\times B_{3}=30^{3}$ box-averaged data points by the data reduction method in Appendix A. However, we found that some of the *B* box-averaged data points were far away from the torus. After ignoring these points, we eventually chose $\tilde{B}=$ 26,020 of the box-averaged data points that were close enough to the torus for training. Then, we trained $K_{1}=1000$ data-driven basis functions on N from these 26,020 box-averaged data points using the VBDM algorithm.

Unlike the previous example, the derivation of the analytical expression for the likelihood function $p(x|D_{j})$ was not trivial. This difficulty is due to the fact that the diffusion coefficient, b(θ,ϕ), is state dependent. While direct MCMC with an approximate likelihood function constructed using the Bayesian imputation [5] can be done in principle, we neglected this numerical computation since the cost in generating the path $\left\{x_{i}\right\}$ for i=1,…,T on each sampling step was too costly in our setup below (where T= 10,000, and we would generate a chain of length 400,000 samples). For diagnostic comparisons, we constructed another representation $\hat{p}\left(x|D_{j}\right)$, named the intrinsic Fourier representation, which can be regarded as an accurate approximation of $p(x|D_{j})$, as it used the basis functions defined on the intrinsic coordinates (θ,ϕ) instead of $x\in R^{3}$. See Appendix B for the construction and the convergence of the intrinsic Fourier representation in detail. We should point out that this intrinsic representation is not available in general since one may not know the embedding of the data manifold.

**Figure 4:**
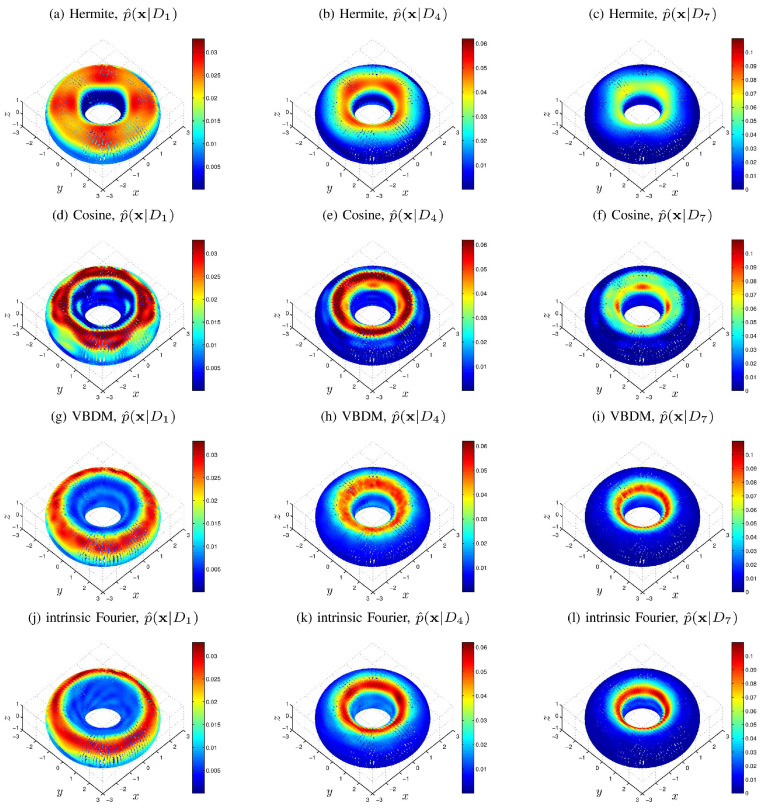
(Color online) Comparison of the conditional densities $\hat{p}\left(x|D_{j}\right)$ estimated by using Hermite representation (first row), cosine representation (second row), VBDM representation (third row), and intrinsic Fourier representation (fourth row). The left (**a**,**d**,**g**,**j**), middle (**b**,**e**,**h**,**k**), and right (**c**,**f**,**i**,**l**) columns correspond to the densities on the training parameters $D_{1}=0.25$, $D_{4}=1.00$, and $D_{7}=1.75$, respectively. $K_{1}=1000$ basis functions are used for all representations. For fair visual comparison, all conditional densities are plotted on the same box-averaged data points and normalized to satisfy $\frac{1}{\tilde{B}}\sum_{b=1}^{\tilde{B}}\hat{p}\left(x_{b}|D_{j}\right)/\bar{q}\left(x_{b}\right)=1$ with $\bar{q}$ being the estimated sampling density of the box-averaged data ${\left\{x_{b}\right\}}_{b=1}^{\tilde{B}}$.

Figure 4 displays the comparison of the density estimates. It can be observed from Figure 4 that the VBDM representation was in good agreement with the intrinsic Fourier representation, whereas Hermite and cosine representations of $\hat{p}\left(x|D_{j}\right)$ deviated significantly from the intrinsic Fourier representation. The reason in short was that if the density p(θ,ϕ|D) in (θ,ϕ) coordinate were in $H\left({\left[0,2\pi \right)}^{2}\right)\subset L^{2}\left({\left[0,2\pi \right)}^{2}\right)$, then the corresponding VBDM representation with respect to dV(x) would be in $H_{{\bar{q}}^{-1}}\left(N\right)$. However, the representation (Hermite and cosine) with respect to dx, $x\in R^{3}$ is not in $H_{{\bar{q}}^{-1}}\left(R^{3}\right)$. A more detailed explanation of this assertion is presented in Appendix B.

**Figure 5:**
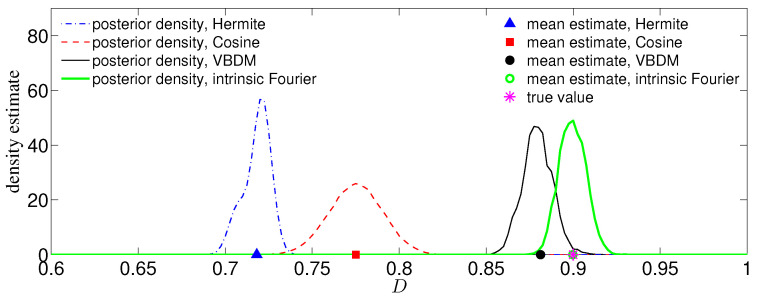
(Color online) Comparison of the posterior density functions by all representations. Plotted also are mean estimates by Hermite representation $\hat{D}=0.78$ (blue triangle), cosine representation $\hat{D}=0.79$ (red square), VBDM representation $\hat{D}=0.88$ (black circle), the intrinsic Fourier representation $\hat{D}=0.90$ (green circle), and the true parameter value $D^{†}=0.9$ (magenta asterisk).

We now compare the MCMC estimates with the true value, $D^{†}=0.9$, from T= 10,000 observations. For this simulation, we set the prior to be uniformly distributed and empirically chose C=0.01 for the proposal. Figure 5 displays the posterior densities of the chains for all representations (each plot of the density estimate was constructed using KDE on a chain of length 400,000). Displayed also is the comparison between the true value $D^{†}$ and the MCMC mean estimates by all representations. Here, the mean estimate by the intrinsic Fourier representation nearly overlaps with the true value $D^{†}=0.9$, as shown in Figure 5. The mean estimate by the VBDM representation is closer to the true value $D^{†}$ compared to the estimates by Hermite and cosine representations. Moreover, it can be seen from Figure 5 that the posterior by the VBDM representation is close to the posterior by intrinsic Fourier representation, whereas the posterior densities by Hermite and cosine representation are not. We should point out that this result is encouraging considering that the training parameter domain is rather wide, $D_{j}\in \left[1/4,2\right]$. This result suggests that when the intrinsic dimension is less than the ambient space dimension, d<n, the VBDM representation (which does not require the knowledge of the embedding function in (55)) with data-driven basis functions in $L^{2}\left(N,\bar{q}\right)$ is superior compared to the representations with analytic basis functions defined on the ambient coordinates $R^{3}$.

### 5.3. Example III: Five-Dimensional Lorenz-96 Model

Consider the Lorenz-96 model [28]:

(56) $$\frac{dx_{j}}{dt}=x_{j-1}\left(x_{j+1}-x_{j-2}\right)-x_{j}+F,\;\;\;j=1,\ldots ,J,$$

with periodic boundary, $x_{j+J}=x_{j}$. For the example in this section, we set J=5. The initial condition was $x_{j}\left(0\right)=\sin\left(2\pi j/5\right)$. Our goal here was to estimate the posterior density and posterior mean of the hidden parameter *F* given a time series of noisy observations $y^{†}=\left(y_{1}^{†},y_{2}^{†},y_{3}^{†},y_{4}^{†},y_{5}^{†}\right)$, where:

$$y_{j}^{†}\left(t_{m}\right)=x_{j}^{†}\left(t_{m}\right)+\epsilon_{m,j},\;\;\;\epsilon_{m,j}∼N\left(0,\sigma^{2}\right),\;\;m=1,\ldots ,T,$$

with noise variance $\sigma^{2}=0.01$. Here, $x_{j}^{†}\left(t_{m}\right)$ denotes the approximate solution (with the Runge–Kutta method) with a specific parameter value $F^{†}$ at discrete times $t_{m}=ms▵t$, where ▵t=0.05 is the integration time step and *s* is the observation interval. Since the embedding function of the observation data is unknown, we do not have a parametric analog to the intrinsic Fourier representation as in the previous example.

In this low-dimensional setting, we can compare the proposed method with basic techniques, including the direct MCMC and the Non-Intrusive Spectral Projection (NISP) method [13]. By direct MCMC, we refer to employing the random walk Metropolis scheme directly on the following likelihood function,

(57) $$p\left(y^{†}|F\right)\propto \exp\left(-\frac{\sum_{m=1}^{T}\sum_{j=1}^{5}{\left(y_{j}^{†}\left(t_{m}\right)-x_{j}\left(t_{m};F\right)\right)}^{2}}{2\sigma^{2}}\right),$$

where $\sigma^{2}$ is the noise variance and $x_{j}\left(t_{m};F\right)$ is the solution of the initial value problem in Equation (56) with the parameter *F* at time $t_{m}$. Note that evaluating $x_{j}\left(t_{m};F\right)$ is time consuming if the model time TsΔt is long or the MCMC chain has many iterations. In our implementation, we generated the chain for 4000 iterations. This amounts to 4000 sequential evaluations of the likelihood function in (57), where each evaluation requires integrating the model in (56) with the proposal parameter value $F^{*}$ until model unit time TsΔt. We used a uniform prior distribution and C=0.1 for the proposal.

For the NISP method [13], we used the same Gaussian likelihood function (57) with approximated $x_{j}$. In particular, we approximated the solutions $x_{j}$ with ${\tilde{x}}_{j}\left(t,F\right)$ for j=1,…,5 in the form of:

(58) $${\tilde{x}}_{j}\left(t,F\right)=\sum_{k=1}^{K}{\hat{x}}_{j,k}\left(t\right)\varphi_{k}\left(F\right),$$

where $\varphi_{k}\left(F\right)$ are chosen to be the orthonormal cosine basis functions, ${\hat{x}}_{j,k}\left(t\right)$ are the expansion coefficients, and *K* is the number of basis functions. Subsequently, we prescribe a fixed set of nodes ${\left\{F_{j}=7.55+0.1j\right\}}_{j=1}^{8}$ to be used for training ${\hat{x}}_{j,k}\left(t\right)$. Practically, this training procedure only requires eight model evaluations that can be done in parallel, where each evaluation involves integrating the model with the specified $F_{j}$ until model unit time TsΔt. The number of basis functions is K=8. After specifying the coefficients ${\hat{x}}_{j,k}\left(t\right)$ such that ${\tilde{x}}_{j}\left(t,F\right)=x_{j}\left(t;F\right)$, we obtain the approximation of the solutions ${\tilde{x}}_{j}\left(t,F\right)$ for all parameters *F*. Using these approximated ${\tilde{x}}_{j}\left(t,F\right)$, in place of $x_{j}\left(t_{m},F\right)$ in (57), we can generate the Markov chain using the Metropolis scheme. Again, we used a uniform prior distribution and C=0.1 for the proposal. In our MCMC implementation, we generated the chain for 40,000 iterations; this involved only evaluating (58) instead of integrating the true dynamical model in (56) on the proposal parameter value $F^{*}$.

For RKWHS representations, we also used M=8 uniformly-distributed training parameters, ${\left\{F_{j}=7.55+0.1j\right\}}_{j=1}^{8}$. As in the NISP, this training procedure required only eight model integrations with parameter value $F_{j}$ until the model unit time TsΔt, resulting in a total of MN=8Ts training data. In this example, we did not reduce the data using the box-averaging method in Appendix A. In fact, for some cases, such as s=1 and T=50, the total of training data were only MN=400, which was too few for estimation of the eigenfunctions. Of course, one can consider more training parameters to increase this training dataset, but for a fair comparison with NISP, we chose to just add 10 i.i.d. Gaussian noises to each dataset, resulting in a total of MN=4000 for training dataset. This configuration (with a small dataset) is a tough setting for the VBDM since the nonparametric method is advantageous in the limit of a large dataset. When 8Ts is sufficiently large, we do not need to increase the dataset by adding multiple i.i.d Gaussian noises.

For Hermite and Cosine representation, we constructed five Hermite and cosine basis functions for each coordinate, which yielding a total of $K_{1}=5^{5}=3125$ basis functions in $R^{5}$. For the VBDM representation, we directly applied the VBDM algorithm to train $K_{1}=3125$ data-driven basis functions on manifold N from the MN=4000 training dataset. From the VBDM algorithm, the estimated intrinsic dimension was d≈2, which was smaller than the dimension of the ambient space n=5. Then, we applied a uniform prior distribution and C=0.01 for the proposal. As in NISP, we generated the chain for 40,000 iterations, which amounted to evaluating (44) instead of integrating the true dynamical model in (56) on each iteration.

**Figure 6:**
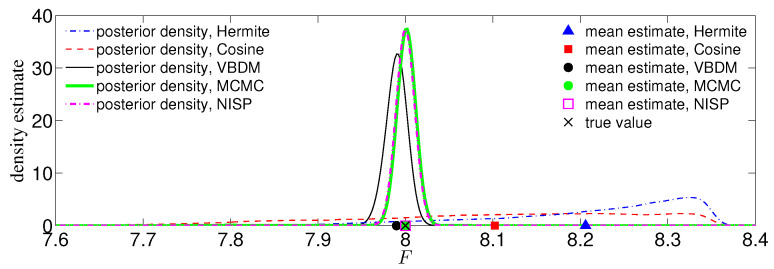
(Color online) Comparison of the posterior density functions among the direct MCMC method, NISP method, and all RKWHS representations. Plotted also are the true parameter value $F^{†}=8$ (black cross), mean the estimate by the direct MCMC method $\hat{F}=8.00$ (green circle), the mean estimate by the NISP method $\hat{F}=8.00$ (magenta square), and the mean estimates by Hermite representation $\hat{F}=8.21$ (blue triangle), cosine representation $\hat{F}=8.10$ (red square), and VBDM representation $\hat{F}=7.99$ (black circle). The noisy observations are $y_{j^{†}}\left(t_{m}\right)$ for s=1, T=50.

We now compare the posterior densities and mean estimates for the case of s=1 and T=50 noisy observations $y^{†}\left(t_{m}\right)$ corresponding to the true parameter value $F^{†}=8$. Figure 6 displays the posterior densities of the chains and mean estimates for the direct MCMC method, NISP method, and all representations. It can be seen from Figure 6 that the mean estimate by VBDM representation was in good agreement with the true value $F^{†}$. In contrast, the mean estimates by Hermite and cosine representations deviated substantially from the true value. Based on this numerical result, where the estimated intrinsic dimension d≈2 of the observations was lower than the ambient space dimension n=5, the data-driven VBDM representation was superior compared to the Hermite and cosine representations. It can be further observed that direct MCMC, NISP, and VBDM representation can provide good mean estimates to the true value. However, notice that we only ran the model M=8 times for the NISP method and VBDM representation, whereas we ran the model 4000 times for the direct MCMC method.

**Figure 7:**
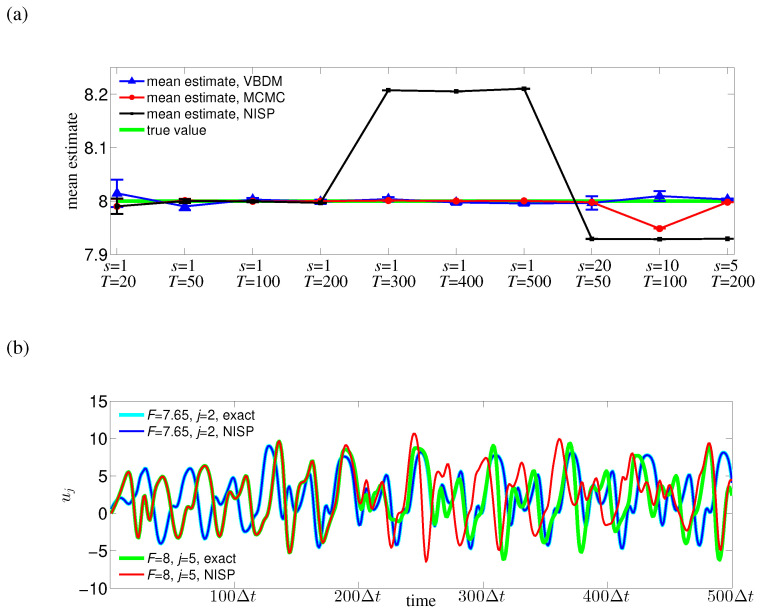
(Color online) (**a**) Comparison of the mean estimates among the direct MCMC method, NISP method, and VBDM representation for different cases of *s* and *T*. Plotted also is the true parameter value $F^{†}=8$ (green curve). (**b**) Comparison of the exact solution by numerical integration and the approximated solution by the NISP method at the training parameter F=7.65 and at the parameter value F=8, which is not in the training parameter.

In real applications where the observations are not simulated by the model, we expect the observation configuration to be pre-determined. Therefore it is important to have an algorithm that is robust under various observation configurations. In our next numerical experiment, we checked such robustness by comparing the direct MCMC method, NISP method, and VBDM representation for different cases of *s* and *T* (Figure 7a). It can be observed from Figure 7a that both the direct MCMC method and VBDM representation can provide reasonably accurate mean estimates for all cases of *s* and *T*. However, again notice that we need to run the model much more times for the direct MCMC method than for VBDM representation. It can be further observed that the NISP method can only provide a good mean estimate for observation time up to TsΔt=200Δt when eight uniform nodes ${\left\{F_{j}=7.55+0.1j\right\}}_{j=1}^{8}$ are used. The reason was that the approximated solution by NISP method was only accurate for observation time up to 200Δt (see the green and red curves in Figure 7b). This result suggests that our surrogate modeling approach using the VBDM representation can provide accurate and robust mean estimates under various observation configurations.

### 5.4. Example IV: The 40-Dimensional Lorenz-96 Model

In this section, we consider estimating the parameter *F* in the Lorenz-96 model in (56), but of a J=40 dimensional system. We now consider observing the autocorrelation function of several energetic Fourier modes of the system phase-space variables. In particular, let ${\left\{{\hat{x}}_{k}\left(t_{m};F\right)\right\}}_{k=-J/2+1,\ldots ,J/2}$ be the $k^{\mathrm{th}}$ discrete Fourier mode of ${\left\{x_{j}\left(t_{m};F\right)\right\}}_{k=1\ldots ,J}$, where $t_{m}=m\Delta t$ with Δt=0.05. Let the observation function be defined as in (4) with four-dimensional ${\left\{y_{m}\left(F\right)\right\}}_{m=0,\ldots ,T}$, whose components are the autocorrelation function of Fourier mode $k_{j}$,

$$\begin{array}{l} y_{m,j}\left(F\right)=E\left[{\hat{x}}_{k_{j}}\left(t_{m};F\right){\hat{x}}_{k_{j}}\left(t_{0};F\right)\right],\;m=0,\ldots ,T,\;j=1,\ldots ,4, \end{array}$$

of the energetic Fourier modes, $k_{j}\in \left\{7,8,9,14\right\}$. See [29] for the detailed discussion of the statistical equilibrium behavior of this model for various values of *F*. Such observations arise naturally since some of the model parameters can be identified from non-equilibrium statistical information via the linear response statistics [30,31]. In our numerics, we will approximate the correlation function by averaging over a long trajectory,

(59) $$\begin{array}{l} E\left[{\hat{x}}_{k_{j}}\left(t_{m};F\right){\hat{x}}_{k_{j}}\left(t_{0};F\right)\right]\approx \frac{1}{L}\sum_{\ell =1}^{L}{\hat{x}}_{k_{j}}\left(t_{m+\ell };F\right){\hat{x}}_{k_{j}}\left(t_{\ell };F\right), \end{array}$$

with $L=10^{6}$. Here, each of these Fourier modes is assumed to have zero empirical mean. We will consider observing the autocorrelation function up to time T=50 (corresponding to 2.5 unit time).

With this setup, the corresponding likelihood function for $p(y_{m}|F)$ is not easily approximated (since it is not in the form of (6)), and it is computationally demanding to generate $y_{m,j}\left(F\right)$ since each evaluation requires integration of the 40-dimensional Lorenz-96 model up to time index $L=10^{6}$. This expensive computational cost makes either the direct MCMC or approximate Bayesian computation infeasible. We should also point out the fact that a long trajectory is needed in the evaluation of (59), making this problem intractable with NISP even if a parametric likelihood function becomes available. This issue is because the approximated trajectory by polynomial chaos expansion in NISP is only accurate for short times, as shown in the previous example. We will consider constructing the likelihood function from a wide range of training parameter values, $F_{i}=6+0.1\left(i-1\right),i=1,\ldots ,M=31$. This parameter domain is rather wide and includes the weakly chaotic regime (F=6) and strongly chaotic regime (F=8). See [32] for a complete list of chaotic measures in these regimes including the largest Lyapunov exponent and the Kolmogorov–Sinai entropy.

**Figure 8:**
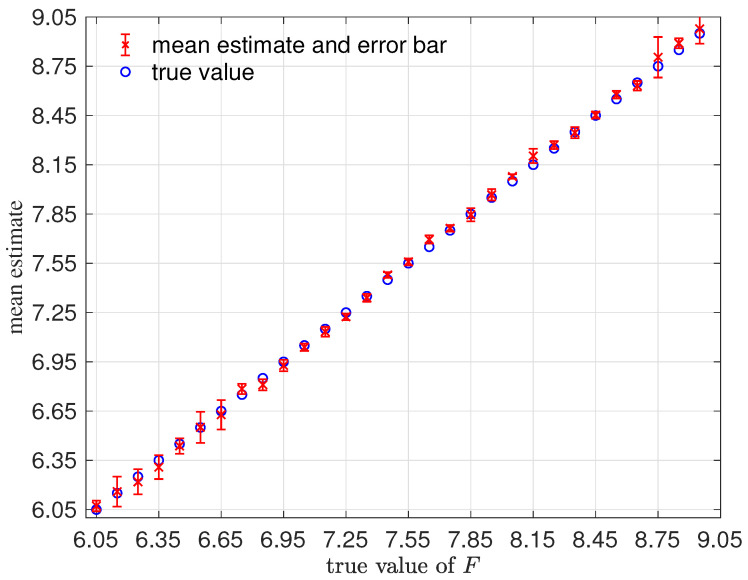
(Color online) Mean error estimates and error bars for various true values of *F* that are not in the training parameters.

In this setup, we had a total of MN=M(T+1)=31×51=1581 of $y_{m}\left(F_{i}\right)\in R^{4}$ for training. We will consider an RKHS representation with $K_{1}=500$ basis functions. We will demonstrate the performance on 30 sets of observations $y_{m}\left(F_{s}^{†}\right)$, where in each case, $F_{s}^{†}$ does not belong to the training parameter set, namely $F_{s}^{†}=6.05+0.1\left(s-1\right),s=1,\ldots ,30$. In each simulation, the MCMC initial chain will be set to be random, F∼U(6.5,8.5); the prior is uniform; and C=0.01 for the proposal. In Figure 8, we show the mean estimates and an error bar (based on one standard deviation) computed from averaging the MCMC chain of length 40,000 in each case. Notice the robustness of these estimates on a wide range of true parameter values $F^{†}$ using a likelihood function constructed using a single set of training parameter values on [6,9].

## 6. Conclusions

We have developed a framework of a parameter estimation approach where MCMC was employed with a nonparametric likelihood function. Our approach approximated the likelihood function using the kernel embedding of conditional distribution formulation based on RKWHS. By analyzing the error estimation in Theorem 1, we have verified the validity of our RKWHS representation of the conditional density as long as $p\left(y|\theta_{j}\right)\in H_{q^{-1}}\left(N\right)$ induced by the basis in $L^{2}\left(N,q\right)$ and Var${}_{Y|\theta_{j}}\left[\psi_{k}\left(Y\right)\right]$ is finite. Furthermore, the analysis suggests that if the weight *q* is chosen to be the sampling density of the data, the Var${}_{Y|\theta_{j}}\left[\psi_{k}\left(Y\right)\right]$ is always finite. This justifies the use of Variable Bandwidth Diffusion Maps (VBDM) for estimating the data-driven basis functions of the Hilbert space weighted by the sampling density on the data manifold.

We have demonstrated the proposed approach with four numerical examples. In the first example, where the dimension of the data manifold was exactly the dimension of the ambient space, d=n, the RKHS representation with VBDM basis yielded a parameter estimate as accurate as using other analytic basis representation. However, in the examples where the dimension of the data manifold was strictly less than the dimension of the ambient space, d<n, only VBDM representation could provide more accurate estimation of the true parameter value. We also found that VBDM representation produced mean estimates that were robustly accurate (with accuracies that were comparable to the direct MCMC) on various observation configurations where the NISP was not accurate. This numerical comparison was based on using only eight model evaluations, which can be done in parallel for both VBDM and NISP, whereas the direct MCMC involved 4000 sequential model evaluations. Finally, we demonstrated robust accurate parameter estimation on an example where the analytic likelihood function was intractable and computationally demanding, even if it became available. Most importantly, this result was based on training on a wide parameter domain that included different chaotic dynamical behaviors.

From our numerical experiments, we conclude that the proposed nonparametric representation was advantageous in any of these configurations: (1) when the parametric likelihood function was not known, such as in Example IV; (2) when the observation time stamp was long (such as in Example II or for large sT in Example III and Example IV). Ultimately, the only real advantage of this method (as a surrogate model) was when the direct MCMC or ABC, which require sequential model evaluation, was computationally not feasible.

While the theoretical and numerical results were encouraging as a proof the concept for using the VBDM representation in many other parameter estimation applications, there were still practical limitations that need to be overcome. As in the other surrogate modeling approaches, one needs to have knowledge of the feasible domain for the parameters. Even when the parameter domain is given and wide, it is practically not feasible to generate training dataset by evaluating the model on the specified training grid points on this domain when the dimension of the parameter space is large (e.g., order 10), even if the Smolyak sparse grid is used. One possible way to simultaneously overcome these two issues is to use “crude” methods, such as ensemble Kalman filtering or smoothing, to obtain the training parameters. We refer to such a method as “crude” since the parameter estimation with ensemble Kalman filtering is sensitive to the initial conditions, especially when the persistent model is used as the dynamical model for the parameters [23]. However, with such crude methods, we can at least obtain a set of parameters that reflect the observational data, instead of specifying training parameters uniformly or in a random fashion, which can lead to unphysical training parameters. Another issue that arises in the VBDM representation is the expensive computational cost when the amount of data MN is large. When the dimension of the observations is low (as in the examples in this paper), the data reduction technique described in Appendix A is sufficient. For larger dimensional problems, a more sophisticated data reduction is needed. Alternatively, one can explore representations using other orthonormal data-driven basis, such as the QR factorized basis functions as a less expensive alternative to the eigenbasis [27].

## Figures and Tables

**Figure A1:**
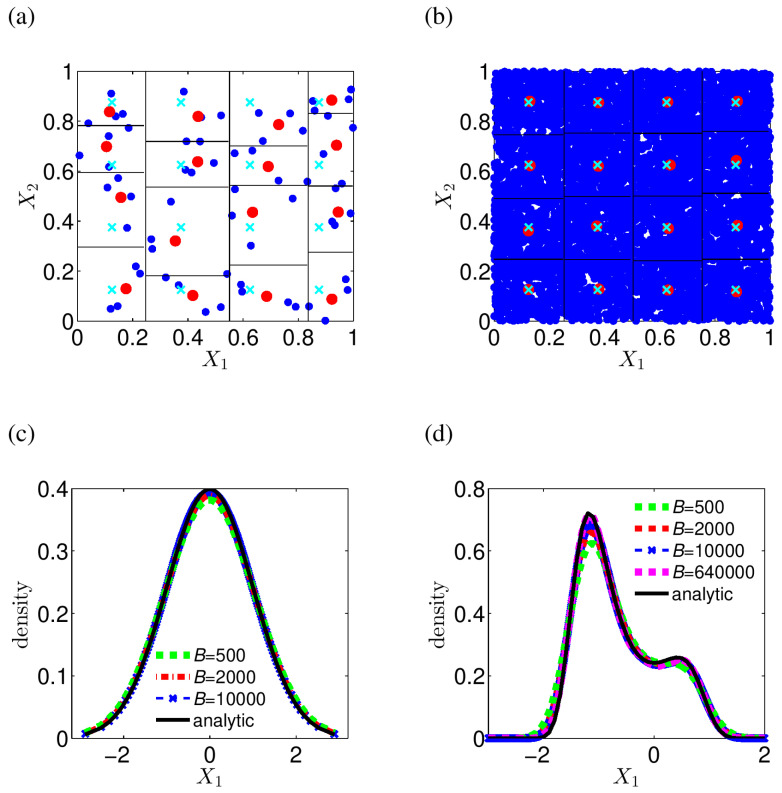
(Color online) Data reduction for (**a**) few number (=64) of uniformly distributed data, and (**b**) many number (=6400) of uniformly distributed data. The 64 blue circles correspond to uniformly distributed data, 16 cyan crosses correspond to well-sampled uniformly distributed data, and 16 red circles correspond to box-averaged data. Boxes are partitioned by horizontal and vertical black lines. The vertical black lines correspond to the first clustering and the horizontal lines correspond to the second clustering. Panels (**c**) and (**d**) display the comparison of kernel density estimates on the box-averaged data for different number *B* for (c) standard normal distribution, and (d) the distribution proportional to $\exp[-\left(-X_{1}^{2}+X_{1}^{3}+X_{1}^{4}\right)]$, respectively. For comparison, also plotted is the analytic probability density of the distribution. The total number of the points is 640,000. It can be seen that the reduced box-averaged data points nearly preserve the distribution of the original dataset.

## Abbreviations

The following abbreviations are used in this manuscript:

VBDM	Variable Bandwidth Diffusion Maps
RKHS	Reproducing Kernel Hilbert Space
RKWHS	Reproducing Kernel Weighted Hilbert Space
MCMC	Markov Chain Monte Carlo
ABC	Approximate Bayesian Computation
NISP	Non-Intrusive Spectral Projection

## Appendix A. Data Reduction

When MN is very large, the VBDM algorithm becomes numerically expensive since it involves solving an eigenvalue problem of matrix size MN×MN. Notice that the number of training parameters *M* grows exponentially as a function of the dimension of parameter, *m*, if well-sampled uniformly distributed training parameters are used. To overcome this large training data problem, we employ an empirical data reduction method to reduce the original MN training data points ${\left\{y_{i,j}\right\}}_{j=1,\ldots ,M}^{i=1,\ldots ,N}$ to a small $B\left(\ll MN\right)$ number of training data points yet preserving the sampling density $\bar{q}\left(y\right)$ in (37). Subsequently, we apply the VBDM algorithm on these reduced training data points. It is worthwhile to mention that this data reduction method is numerically applicable for low-dimensional dataset although in the following we will introduce this reduction method for any *n*-dimensional dataset.

The basic idea of our method is that we first cluster the training dataset ${\left\{y_{i,j}\right\}}_{j=1,\ldots ,M}^{i=1,\ldots ,N}$ into *B* number of boxes and then take the average of data points in each box as a reduced training data point. First, we cluster the training data $\left\{y_{i,j}\right\}$, based on the ascending order of the 1^st^ coordinate of the training data, $\left\{y_{i,j}^{1}\right\}$, into $B^{1}$ number of groups such that each group has the same number $\left(=MN/B^{1}\right)$ of data points. After the first clustering, we obtain $B^{1}$ groups with each group denoted by $G_{k_{1}}^{1}$ for $k_{1}=1,\ldots ,B^{1}$. Here, the super-index 1 denotes the first clustering and the sub-index $k_{1}$ denotes the $k_{1}^{\mathrm{th}}$ group. Second, for each group $G_{k_{1}}^{1}$, we cluster the training data $\left\{y_{i,j}\right\}$ inside $G_{k_{1}}^{1}$, based on the ascending order of the second coordinate of the training data, $\left\{y_{i,j}^{2}\right\}$, into $B^{2}$ number of groups such that each group has the same number $\left(=MN/B^{1}B^{2}\right)$ of data points. After the second clustering, we obtain totally $B^{1}B^{2}$ groups with each group denoted by $G_{k_{1}k_{2}}^{2}$ for $k_{1}=1,\ldots ,B^{1},$ and $k_{2}=1,\ldots ,B^{2}$. We can operate such clustering *n* times, where *n* is the dimension of the observation y ambient space. After *n* times clustering, we obtain $B\equiv \prod_{s=1}^{n}B^{s}$ groups with each group denoted by $G_{k_{1}k_{2}\ldots k_{n}}^{n}$ with $k_{s}=1,\ldots ,B^{s},$ for all s=1,…,n. Each group is a box [see Figure A1 for example]. After taking the average of the data points in each box $G_{k_{1}k_{2}\ldots k_{n}}^{n}$, we obtain *B* number of reduced training data points. In the remainder of this paper, we denote these *B* number of reduced training data points by ${\left\{{\bar{y}}_{b}\right\}}_{b=1,\ldots ,B}$ and refer to them as the box-averaged data points. Intuitively, this algorithm partitions the domain into hyperrectangle such that $P_{r}\left(\bar{y}\in G_{k_{1}\ldots k_{n}}^{n}\right)\approx 1/B$. Note that the idea of our data reduction method is analogous to that of multivariate *k*-nearest neighbor density estimates [33,34]. The error estimation can be found in the Refs. [33,34].

To examine whether the distribution of these box-averaged data points is close to the sampling density $\bar{q}\left(y\right)$ of the original dataset, we apply our reduction method to several numerical examples in the following. Figure A1a shows the reduction result for few number (=64) of uniform data in [0,1]×[0,1]. Here, $B^{1}=4$ and $B^{2}=4$ so that there are total B=16 boxes and inside each box there are 4 uniform data points (blue circles). It can be seen that the box-averaged data points (red circles) are far away from the well-sampled uniform data points (cyan crosses). However, when uniform data points (blue circles) increase to a large number (=6400), box-average data points (red circles) are very close to well-sampled uniform data points (cyan crosses) as shown in Figure A1b. This suggests that these box-averaged data points nearly admit the uniform distribution when there are a large number of original uniform data points. Figure A1c and d show the comparison of the kernel density estimates applied on the box-averaged data for different *B* for standard normal distribution and the distribution proportional to $\exp\left[-\left(-X_{1}^{2}+X_{1}^{3}+X_{1}^{4}\right)\right]$, respectively. It can be seen that the reduced box-averaged data points nearly preserve the distribution of the original large dataset, N=640,000.

When MN is very large, the VBDM algorithm for the construction of data-driven basis functions [Procedure (*1-B*)] can be outlined as follows. We first use our data reduction method to obtain $B\left(\ll MN\right)$ number of box-averaged data ${\left\{{\bar{y}}_{b}\right\}}_{b=1,\ldots ,B}\subseteq N\subseteq R^{n}$ with sampling density $\bar{q}\left(y\right)\approx \frac{1}{M}\sum_{j=1}^{M}p\left(y|\theta_{j}\right)$ in (37). The sampling density $\bar{q}$ is estimated at the box-averaged data ${\bar{y}}_{b}$ using all the box-averaged data points ${\left\{{\bar{y}}_{b}\right\}}_{b=1,\ldots ,B}$ by a kernel density estimation method. Implementing the VBDM algorithm, we can obtain orthonormal eigenvectors ${\bar{\psi }}_{k}\in R^{B}$, which are discrete estimates of the eigenfunctions ${\bar{\psi }}_{k}\left(y\right)\in L^{2}\left(N,\bar{q}\right)$. The $b^{\mathrm{th}}$ component of the eigenvector ${\bar{\psi }}_{k}$ is a discrete estimate of the eigenfunction ${\bar{\psi }}_{k}\left({\bar{y}}_{b}\right)$, evaluated at the box-averaged data point ${\bar{y}}_{b}$. Due to the dramatic reduction of the training data, the computation of these eigenvectors ${\bar{\psi }}_{k}\in R^{B}$ becomes much cheaper than the computation of the eigenvectors ${\bar{\psi }}_{k}\in R^{MN}$ using the original training dataset ${\left\{y_{i,j}\right\}}_{j=1,\ldots ,M}^{i=1,\ldots ,N}$. Then we can obtain a discrete representation (10) of the conditional density at the box-averaged data points ${\bar{y}}_{b}$, $\hat{p}\left({\bar{y}}_{b}|\theta \right)$.

## Appendix B. Additional Results on Example II

In this section, we discuss the intrinsic Fourier representation constructed for numerical comparisons in Example II and provide more detailed discussion on the numerical results.

We first discuss the construction for the intrinsic Fourier representation of the true conditional density, $p\left(x|D\right)$, defined with respect to the volume form inherited by N from the ambient space $R^{n}$ for the system on the torus (54) in Example II. By noticing the embedding (55) of $\left(\theta ,\phi \right)$ in $x\equiv \left(x,y,z\right)$, we can obtain the following equality,

(A1) $$\begin{array}{lll} 1 & = & \int_{N}p\left(x|D\right)dV\left(x\right)=\int_{{\left[0,2\pi \right)}^{2}}p\left(x\left(\theta ,\phi \right)|D\right)\left|x_{\theta }\times x_{\phi }\right|d\theta d\phi \\ & \equiv & \int_{{\left[0,2\pi \right)}^{2}}p_{\mathrm{IC}}\left(\theta ,\phi |D\right)d\theta d\phi , \end{array}$$

where $dV\left(x\right)=\left|x_{\theta }\times x_{\phi }\right|d\theta d\phi$ is the volume form, $p_{\mathrm{IC}}$ denotes the true conditional density as a function of the intrinsic coordinates, $\left(\theta ,\phi \right)$. Assuming that $p_{\mathrm{IC}}\left(\theta ,\phi |D\right)\in H\left({\left[0,2\pi \right)}^{2}\right)\subset L^{2}\left({\left[0,2\pi \right)}^{2}\right)$, and the relation in (A1), we can construct the intrinsic Fourier representation as follows,

(A2) $$\hat{p}\left(x|D\right)=\frac{{\hat{p}}_{\mathrm{IC}}\left(\theta ,\phi |D\right)}{\left|x_{\theta }\times x_{\phi }\right|},$$

where ${\hat{p}}_{\mathrm{IC}}\left(\theta ,\phi |D\right)$ is a RKWHS representation (10) of the conditional density $p_{\mathrm{IC}}\left(\theta ,\phi |D\right)$ with a set of orthonormal Fourier basis functions $\psi_{k}\left(\theta ,\phi \right)\in L^{2}\left({\left[0,2\pi \right)}^{2}\right)$. Here, $\psi_{k}\left(\theta ,\phi \right)$ are formed by the tensor product of two sets of orthonormal Fourier basis functions {1, $\left\{\sqrt{2}\cos\left(m\theta \right)\right\}$, $\left\{\sqrt{2}\sin\left(m\theta \right)\right\}\}$ and {1, $\left\{\sqrt{2}\cos\left(m\phi \right)\right\}$, $\left\{\sqrt{2}\sin\left(m\phi \right)\right\}\}$ for $m\in N^{+}$. Note that for intrinsic Fourier representation, we need to know the embedding (55) and know the data of $\left(\theta ,\phi \right)$ in intrinsic coordinates for training, which is available for this example. Nevertheless, for Hermite, Cosine, and VBDM representations, we only need to know the observation data x for training.

The convergence of $\hat{p}\left(x|D\right)$ to the true density can be explained as follows. For the system (54) in the intrinsic coordinates $\left(\theta ,\phi \right)$, where $p_{\mathrm{IC}}\left(\theta ,\phi |D\right)\in H\left({\left[0,2\pi \right)}^{2}\right)$ for all parameter *D*, the statistics Var${}_{\left(\theta ,\phi \right)|D}\left[\psi_{k}\left(\theta ,\phi \right)\right]$ are bounded for all *D* and all $k\in N^{+}$, by the compactness of ${\left[0,2\pi \right)}^{2}$ and the uniform boundedness of $\psi_{k}\left(\theta ,\phi \right)$ for all *k*. According to Theorem 1, we can obtain the convergence of the representation ${\hat{p}}_{\mathrm{IC}}\left(\theta ,\phi |D\right)$. Then by noticing the smoothness of $\left|x_{\theta }\times x_{\phi }\right|$ on the torus, we can obtain the convergence of the intrinsic Fourier representation $\hat{p}\left(x|D\right)$ in (A2).

Next, we give an intuitive explanation for the reason why in the regime d<n, VBDM representation can provide a good approximation whereas Hermite and Cosine representations cannot. Essentially, the VBDM representation uses basis functions of the weighted Hilbert space of functions defined with respect to a volume form $\tilde{V}$ that is conformally equivalent to the volume form *V* that is inherited by the data manifold N from the ambient space, $R^{n}$. That is, the weighted Hilbert space, $L^{2}\left(N,{\bar{q}}^{-1}\right)$, means,

$$L^{2}\left(N,{\bar{q}}^{-1}\right)=\left\{f:\int_{N}{\left|f\left(x\right)\right|}^{2}d\tilde{V}\left(x\right)<\infty \right\},$$

where $d\tilde{V}\left(x\right)=\bar{q}{\left(x\right)}^{-1}dV\left(x\right)$ denotes the volume form that is conformally changed by the sampling density $\bar{q}$. We should point out that the key point of the diffusion maps algorithm [19] is to introduce an appropriate normalization to avoid biased in the geometry induced by the sampling density $\bar{q}$ when the data are not sampled according to the Riemannian metric inherited by N from the ambient space $R^{n}$. Furthermore, the orthonormal basis functions of the Hilbert space $L^{2}\left(N,{\bar{q}}^{-1}\right)$ are the eigenfunctions of the adjoint (with respect to $L^{2}\left(N\right)$) of the operator, $L=\nabla \log(\bar{q})\cdot \nabla +\Delta$, that is constructed by the VBDM algorithm. Incidentally, the adjoint operator $L^{*}$ is the Fokker-Planck operator of a gradient system forced by stochastic noises. The point is that this adjoint operator takes density functions of the weighted Hilbert space $L^{2}\left(N,{\bar{q}}^{-1}\right)$. Since the Hilbert space $L^{2}\left(N,{\bar{q}}^{-1}\right)$ is a function space of some Fokker-Planck operator that acts on densities defined with respect to the geometry of data, then representing the conditional density with basis functions of the weighted Hilbert space $L^{2}\left(N,{\bar{q}}^{-1}\right)$ is a natural choice. Thus, the error estimation in Theorem 2 is valid in controlling the error of the estimate.

Next, we will show that the representation of the true density $p_{\mathrm{EX}}$ in the ambient space $R^{3}$ is not a function of $H_{q^{-1}}\left(R\right)\subset L^{2}\left(R,q^{-1}\right)$, where for Hermite representation R is $R^{3}$ and *q* is a normal distribution, and for Cosine representation R is a hyperrectangle containing the torus and *q* is a uniform distribution. Recall that the torus is parametrized by:

(A3) $$x\equiv \left(x,y,z\right)=\left(\left(2+r\sin\left(\theta \right)\right)\cos\left(\phi \right),\left(2+r\sin\left(\theta \right)\right)\sin\left(\phi \right),r\cos\left(\theta \right)\right),$$

where θ, ϕ are angles which make a full circle, and *r* is the radius of the tube known as the minor radius. All observation data are located on the torus with r=1. Then, the generalized conditional density p(r,θ,ϕ|D) in $\left(r,\theta ,\phi \right)$ coordinate can be defined using the Dirac delta function as follows,

(A4) $$p\left(r,\theta ,\phi |D\right)=p_{\mathrm{IC}}\left(\theta ,\phi |D\right)\delta \left(r-1\right),$$

where $p_{\mathrm{IC}}$, defined in (A1), denotes the conditional density function in the intrinsic coordinate, $\left(\theta ,\phi \right)$, and δ is the Dirac delta function. After coordinate transformation, the density $p_{\mathrm{EX}}:R^{3}\to R$ can be obtained as

(A5) $$p_{\mathrm{EX}}\left(x|D\right)=\frac{p\left(r,\theta ,\phi |D\right)}{J}=\frac{p_{\mathrm{IC}}\left(\theta ,\phi |D\right)\delta \left(r-1\right)}{J},$$

where *J* is the Jacobian determinant $\det\left[\partial \left(x,y,z\right)/\partial \left(r,\theta ,\phi \right)\right]$. It can be examined that $p_{\mathrm{EX}}\left(x|D\right)$ is a generalized conditional density, that is, $\int_{R^{3}}p_{\mathrm{EX}}\left(x|D\right)dx=1$. Now it can be clearly seen that due to the Dirac delta function $\delta \left(r-1\right)$ in (A5), the density $p_{\mathrm{EX}}\left(x|D\right)$ is no longer in the weighted Hilbert space, $p_{\mathrm{EX}}\left(x|D\right)\notin H_{q^{-1}}\left(R\right)$. Consequently, the error estimation in Theorem 1 becomes invalid in controlling the error of the conditional density.

Here, the key point is that for Cosine and Hermite representations, the volume integral is with respect to dx. The complete basis functions are obtained from tensor product of three sets of basis functions in (x,y,z) coordinates. In order to represent a conditional density function $p_{\mathrm{EX}}\left(x|D\right)$ defined only on an intrinsically 2D torus domain, theoretically infinite number of basis functions are needed. However, numerically only finite number of basis functions can be used. Then, the density $p_{\mathrm{EX}}\left(x|D\right)$ in (A5) cannot be well approximated for Hermite and Cosine representations (10). Moreover, if only finite number of Hermite or Cosine basis functions are used for representations, typically Gibbs phenomenon can be observed, i.e., the Dirac delta function $\delta \left(r-1\right)$ in (A5) will be approximated by a function having a single tall spike at r=1 with some oscillations at two sides along the *r* direction. On the other hand, the data-driven basis functions obtained via the diffusion maps algorithm are smooth functions defined on the data manifold N. Therefore, while the Gibbs phenomenon still occurs in this spectral expansion, it is due to finite truncation in representing a positive smooth functions (densities) on the data manifold, and not due to the singularity that occurs in the ambient direction as in (A5).
